# Identification of the Key Factors Related to Bladder Cancer by lncRNA-miRNA-mRNA Three-Layer Network

**DOI:** 10.3389/fgene.2019.01398

**Published:** 2020-01-28

**Authors:** Xiaxia Wang, Yanrui Ding, Jie Wang, Yanyan Wu

**Affiliations:** ^1^ School of Science, Jiangnan University, Wuxi, China; ^2^ Laboratory of Media Design and Software Technology, Jiangnan University, Wuxi, China; ^3^ Key Laboratory of Industrial Biotechnology, Jiangnan University, Wuxi, China

**Keywords:** bladder cancer, three-layer network, topology, miRNA, TCGA database

## Abstract

Bladder cancer is the most common malignant tumor of the urinary system, and it has high incidence, high degree of malignancy, and easy recurrence after surgery. The etiology and pathogenesis of bladder cancer are not fully understood, but more and more studies have shown that its development may be regulated by some core molecules. To identify key molecules in bladder cancer, we constructed a three-layer network by merging lncRNA-miRNA regulatory network, miRNA-mRNA regulatory network, and lncRNA-mRNA coexpression network, and further analyzed the topology attributes of the network including the degree, betweenness centrality and closeness centrality of nodes. We found that miRNA-93 and miRNA-195 are controllers for a three-layer network and regulators of numerous target genes associated with bladder cancer. Functional enrichment analysis of their target mRNAs revealed that miRNA-93 and miRNA-195 may be closely related to bladder cancer by disturbing the homeostasis of the cell cycle or HTLV-I infection. In addition, since E2F1 and E2F2 are enriched in various KEGG signaling pathways, we conclude that they are important target genes of miRNA-93, and participate in the apoptotic process by forming a complex with a certain protein or transcription factor activity, sequence-specific DNA binding in bladder cancer. Similarly, AKT3 is an important target gene of miRNA-195, its expression is associated with PI3K-Akt-mTOR signaling pathway and AMPK-mTOR signaling pathway. Therefore, we speculate that AKT3 may participate in proliferation and apoptosis of bladder cancer cells through these pathways, and ultimately affect the biological behavior of tumor cells. Furthermore, through survival analysis, we found that miRNA-195 and miRNA-93 are associated with poor prognosis of bladder cancer. And the Kaplan-Meier curve showed that 24 mRNAs and nine lncRNAs are closely related to overall survival of bladder cancer.

## Introduction

Bladder cancer (BC) is a common malignant tumor with the tenth incidence rate and the ninth mortality rate in the world (Bray et al., 2018). As the most common malignant tumor of the urinary system, its etiology and pathogenesis are still not very clear. It has some biological characteristics such as high incidence, high degree of malignancy and easy recurrence after surgery, and it shows a clear upward trend, which is a serious threat to human health and social development (Knollman et al., 2015; Tian et al., 2015). The etiology of BC is complex, including both intrinsic genetic factors and external environmental factors. At present, it has been clarified that the occurrence and development of BC are related to smoking, environmental pollution, and other factors (Kiriluk et al., 2012; Van Roekel et al., 2013; Smith et al., 2016; Wilcox et al., 2016). But its molecular mechanism is still not completely clear. Therefore, the research and clarification of its cause of disease and the specific molecular mechanism will effectively promote the diagnosis and treatment of BC.

In recent years, a large number of studies on single molecules of BC have found that dysregulated mRNA contributes to the occurrence of BC tumors by participating in abnormal activation of many pathways such as Akt signal pathway and NF-kB pathway (Jiang et al., 2016; Tsui et al., 2018). Abnormal expression of lncRNA UCA1 and H19 is closely related to the initiation, development, and metastasis of BC (Zhang et al., 2013). Studies on the relationship between urinary tumors and miR-145, miR-205 indicate that a large number of miRNAs have been involved in tumor progression (Catto et al., 2011; Simonato et al., 2013; Braicu et al., 2015; Chen et al., 2017). Some people also analyzed the differentially expressed miRNAs and their target genes in BC from the perspective of the network, and it is pointed out that miRNAs participate in the occurrence and development of BC by regulating their target genes, and are related to BC recurrence (Guancial et al., 2014; Jin et al., 2015; Li et al., 2017; Zhao et al., 2017). LncRNAs and mRNAs can form complex coexpression networks in various diseases including BC, and promote the development of BC by regulating protein-coding genes (Cao et al., 2018b; Li et al., 2018; Chen et al., 2019a; Chen et al., 2019b). In addition, the ceRNA hypothesis suggests that lncRNA can not only directly participate in the expression of target regulatory genes, but also may adsorb target miRNAs, affect the binding of miRNAs to mRNAs, and further intervene in mRNA expression (Wang et al., 2016; Lv et al., 2017; Wang et al., 2017; Miao et al., 2019; Teng et al., 2019).

Since the mRNA-lncRNA coexpression network and the miRNA-related regulatory network are in the common system, it is one-sided to study the key molecules of BC by the single-layer network method. In contrast, converged network analysis can reflect the importance of molecules in the network more accurately and more directly. Therefore, it is considered as a feasible method to further analyze the topology of the network according to the degree, betweenness centrality and closeness centrality of the nodes in the network to find and predict the core molecules potentially related to the development of BC (Greenbury et al., 2010). In this paper, we constructed a three-layer network by merging lncRNA-miRNA regulatory network, miRNA-mRNA regulatory network and lncRNA-mRNA coexpression network, and further analyzed the topology attributes of nodes in the network.

## Materials and Methods

**Figure 1:**
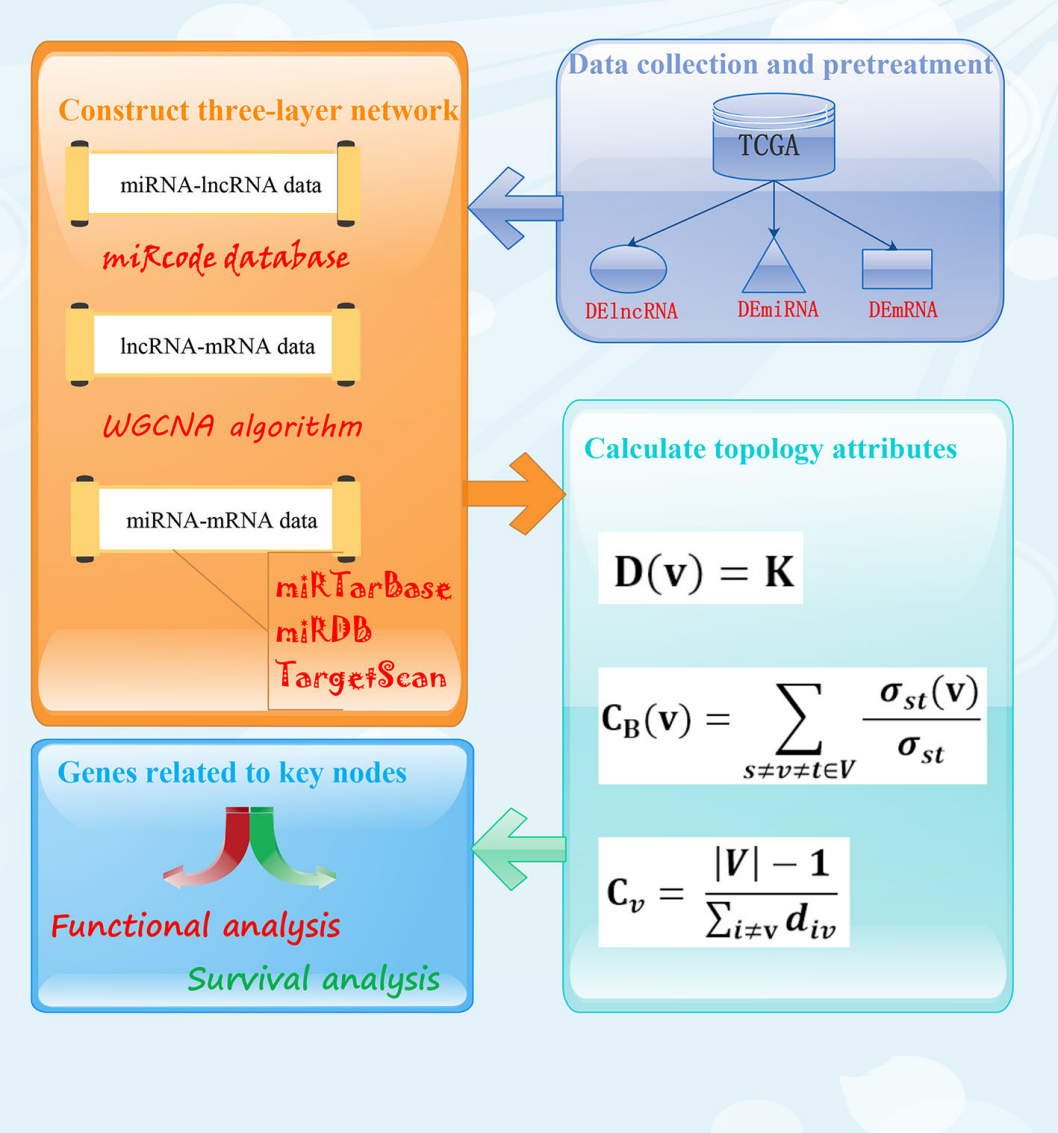
The flowchart of data collection and method implementation.

The main steps of identification of the key factors related to BC by lncRNA-miRNA-mRNA three-layer network include: data collection and pretreatment, construct three-layer network, calculate topology properties, identify the key nodes, functional analysis, and survival analysis of the key nodes. The flowchart of data collection and method implementation was shown in **Figure 1**. Next, we will describe a more detailed process of identification of the key factors related to BC by lncRNA-miRNA-mRNA three-layer network.

### Data Resources and Pretreatment

RNA-Seq data, miRNA-Seq data, and clinical information of transitional cell papillomas and carcinomas in human BC were downloaded from the Cancer Genome Atlas (TCGA) database (https://portal.gdc.cancer.gov/) at the time of 2018-12-03. The RNA-Seq data includes lncRNA and mRNA, including 19 normal samples and 411 BC samples. The miRNA-Seq data contains 19 normal samples and 415 BC samples. We selected their common sample number, 19 normal samples and 405 BC samples, including 19645 mRNAs, 14176 lncRNAs, and 1881 miRNAs.

### Identification of DERNAs

We used “EdgeR” package in R (version 3.5.1) (https://www.r-project.org/) software to identify differentially expressed mRNAs (DEmRNAs), differentially expressed lncRNAs (DElncRNAs) and differentially expressed miRNAs (DEmiRNAs) in BC with the thresholds of Fold change (FC) > 1.5 and adjusted P-value < 0.05.

### Construction of the Three-Layer Network

The target lncRNAs of miRNAs were obtained from miRcode database (http://www.mircode.org/). And miRDB (http://www.mirdb.org/), miRTarBase (http://mirtarbase.mbc.nctu.edu.tw/), and TargetScan database (http://www.targetscan.org/) were used to predict the target mRNAs of miRNAs. Then, the intersection of the target mRNAs predicted by above three databases and DEmRNAs was considered as final target mRNAs of DEmiRNAs.

Using R software, the DEmRNAs and DElncRNAs coexpression data were integrated into a matrix. Then, the lncRNA-mRNA interactions were predicted according to the method of weighted gene coexpression network by using the WGCNA package in R and the correlation coefficient is set to 0.8.

Finally, we merged all miRNA-mRNA pairs and miRNA-lncRNA pairs and mRNA-lncRNA pairs to construct a converged network. We defined the miRNA, mRNA, and lncRNA as the nodes of the network. If they interacted each other, we added an edge between them, then the converged three-layer network was constructed. Cytoscape software (https://cytoscape.org/) was used to visualize the network.

### Calculation of Topology Attributes

Further calculate the topology attribute of the network according to the degree, betweenness centrality, and closeness centrality of the nodes in the three-layer network. For a given graph, G = (V, E), V and E represent a set of nodes and edges, respectively, and degree (D) is usually used to reflect the basic topology attributes of the network, indicating the number of nodes connected to neighboring nodes, assuming K is the number of edges connected to node v, and described as follows:

D(v)=K

The betweenness centrality is one of the most effective methods for evaluating the importance of a node in the network. It is defined as the proportion of the shortest path through a node in the network to the total number of shortest paths, reflecting the influence of the node in the network. It plays an important role in the process of information transfer. And it can be seen from the definition that if a node is the only way for other nodes in the network to communicate, the node has an important position in the network. The higher the node betweenness centrality value, the greater its influence, the expression is as follows:

$$C_{B}\left(v\right)=\underset{s\neq v\neq t\in V}{\sum }\frac{\;\;\sigma_{st}\left(v\right)}{\sigma_{st}}$$

where ‘*σ_st_* (v)’ represents the number of the shortest path between nodes ‘s’ and ‘t’ passing through node ‘v,’ and ‘*σ_st_*’ represents the number of shortest paths between node pairs ‘s’ and ‘t.’

Closeness centrality reflects the proximity of a node in the network to other nodes. Closeness centrality of a node ‘v’ is the reciprocal of the average shortest path distance from node ‘v’ to all other nodes. That is, the closer a node is to other nodes, the greater its closeness centrality. Described as follows:

$$C_{v}=\;\frac{\left|V\right|-1}{\sum_{i\neq v}d_{iv}}\;,$$

where ‘*d_iv_*’ is the shortest-path distance between ‘i’ and ‘v,’ and ‘|V|’ is the number of nodes in the network.

### Extract a Subnetwork From Three-Layer Network

Find important nodes whose topological attributes including degree, betweenness centrality, and closeness centrality ranked in the top ten in the network, namely hub nodes. Then extract the hub nodes and their directly connected RNAs in a three-layer network, and construct a subnetwork related to hub nodes.

### Enrichment Analysis

To achieve enrichment analysis of candidate genes in BC, we used DAVID 6.8(https://david.ncifcrf.gov/) for gene ontology(GO) analysis, including biological process (BP) analysis, cellular component (CC) analysis, and molecular function (MF) analysis, with p < 0.05 as a screening condition to find significantly enriched GO terms. Then, in order to more fully understand the situation of pathway enrichment, KOBAS 3.0 (http://kobas.cbi.pku.edu.cn/) was used as the metabolic pathway KEGG enrichment analysis, at P < 0.01, the number of genes enriched in the pathway was greater than or equal to 2 as a screening condition. By analyzing the significantly enriched GO biological process classification and KEGG pathways, we can further predict the biological function of DEmRNAs from gene function and pathway function, respectively.

### Survival Analysis

According to the clinical information downloaded from TCGA database and the expression level of differentially expressed RNAs, the median was used as the cut-off point, and the RNAs expression was divided into two groups: high expression and low expression. Then, we performed Kaplan-Meier survival analysis on the differentially expressed RNAs, the RNAs in the three-layer converged network, and the RNAs in the subnetwork. The “survival” Package in R software was used to plot the survival curves and view RNAs that is significantly associated with the patient’s survival prognosis. P-value < 0.05 was considered statistically significant.

## Results

### Differentially Expressed RNA

**Figure 2:**
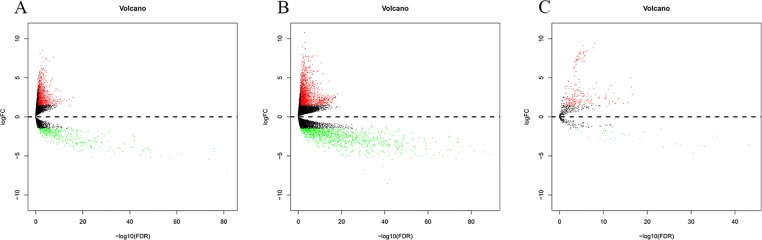
Differentially expressed RNA in bladder cancer (|logFC| > 1.5 and P-value < 0.05). The Panels **(A–C)** are the volcano plot of 1457 DElncRNAs, 3047 DEmRNAs **(B)** and 247 DEmiRNAs. The red denotes upregulations and green denotes downregulations. The x-axis stands for the value of –log10(FDR) and the y-axis stands for the value of logFC.

By comparing normal and cancer samples of BC, 1,457(1,036 upregulated, 421 downregulated, **Figure 2A**) DElncRNAs, 3,047(1,823 upregulated, 1224 downregulated, **Figure 2B**) DEmRNAs, and 247(214 upregulated, 33 downregulated, **Figure 2C**) DEmiRNAs were obtained.

### Construction of a Three-Layer Network

The lncRNA-mRNA coexpression network included 75 lncRNAs, 169 mRNAs, and 380 edges. The lncRNA-miRNA regulatory network constructed in the present study exhibited 129 lncRNAs and 35 miRNAs and 882 edges. And the regulatory network between miRNAs and target genes was established, which included 201 mRNAs, 56 miRNAs, and 487 edges.

**Figure 3:**
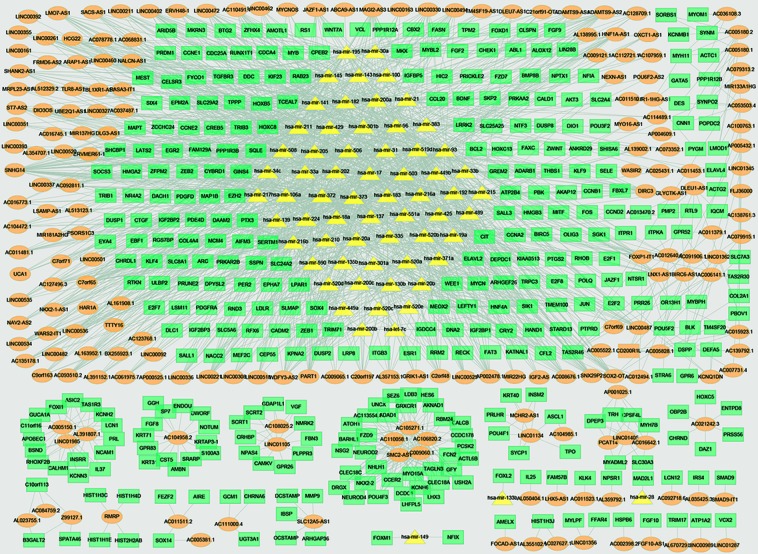
Three-layer network in bladder cancer. Orange ellipse denotes DElncRNAs, green rectangle denotes DEmRNAs, and yellow triangle denotes DEmiRNAs.

Then, we constructed a three-layer network by merging lncRNA-miRNA regulatory network, miRNA-mRNA regulatory network, and lncRNA-mRNA coexpression network, which included 367 mRNAs, 65 miRNAs, 197 lncRNAs, and 1749 edges. The three-layer network was visualized with Cytoscape software and was shown in **Figure 3**.

### Analysis of Topology Attributes

Further analyze the topology of the network and find out the important nodes in the network according to the degree, closeness centrality, and betweenness centrality of the nodes. There are two miRNAs (miRNA-93 and miRNA-195) appeared in each dimension, indicating that they have higher degree, betweenness centrality, and closeness centrality and played crucial roles in the three-layer network. The topology attributes of the top 10 genes were shown in **Table 1**.

**Table 1:** The top 10 genes in degree, betweenness centrality, and closeness centrality. Genes that appear in each column are shown in bold texts.

Gene	Degree	Gene	Betweenness centrality	Gene	Closeness centrality
**hsa-mir-93**	77	**hsa-mir-93**	18,473.228	SNHG14	0.26729
**hsa-mir-195**	67	**hsa-mir-195**	17,484.967	**hsa-mir-93**	0.25661
hsa-mir-17	56	FOXP1-IT1	10,830.214	MAGI2-AS3	0.24637
hsa-mir-106a	54	hsa-mir-211	7,355.569	FOXP1-IT1	0.24375
hsa-mir-211	52	hsa-mir-21	7,301.496	**hsa-mir-195**	0.24228
hsa-mir-519d	51	SNHG14	7,076.982	LINC00472	0.24083
hsa-mir-373	48	hsa-mir-182	6,246.760	SNX29P2	0.23868
hsa-mir-182	45	hsa-mir-200a	6,215.666	ADAMTS9-AS2	0.23727
hsa-mir-372	45	hsa-mir-19a	6,010.876	hsa-mir-106a	0.23518
hsa-mir-145	42	hsa-let-7c	5,751.381	LINC00520	0.23313

### Functional Analysis

**Figure 4:**
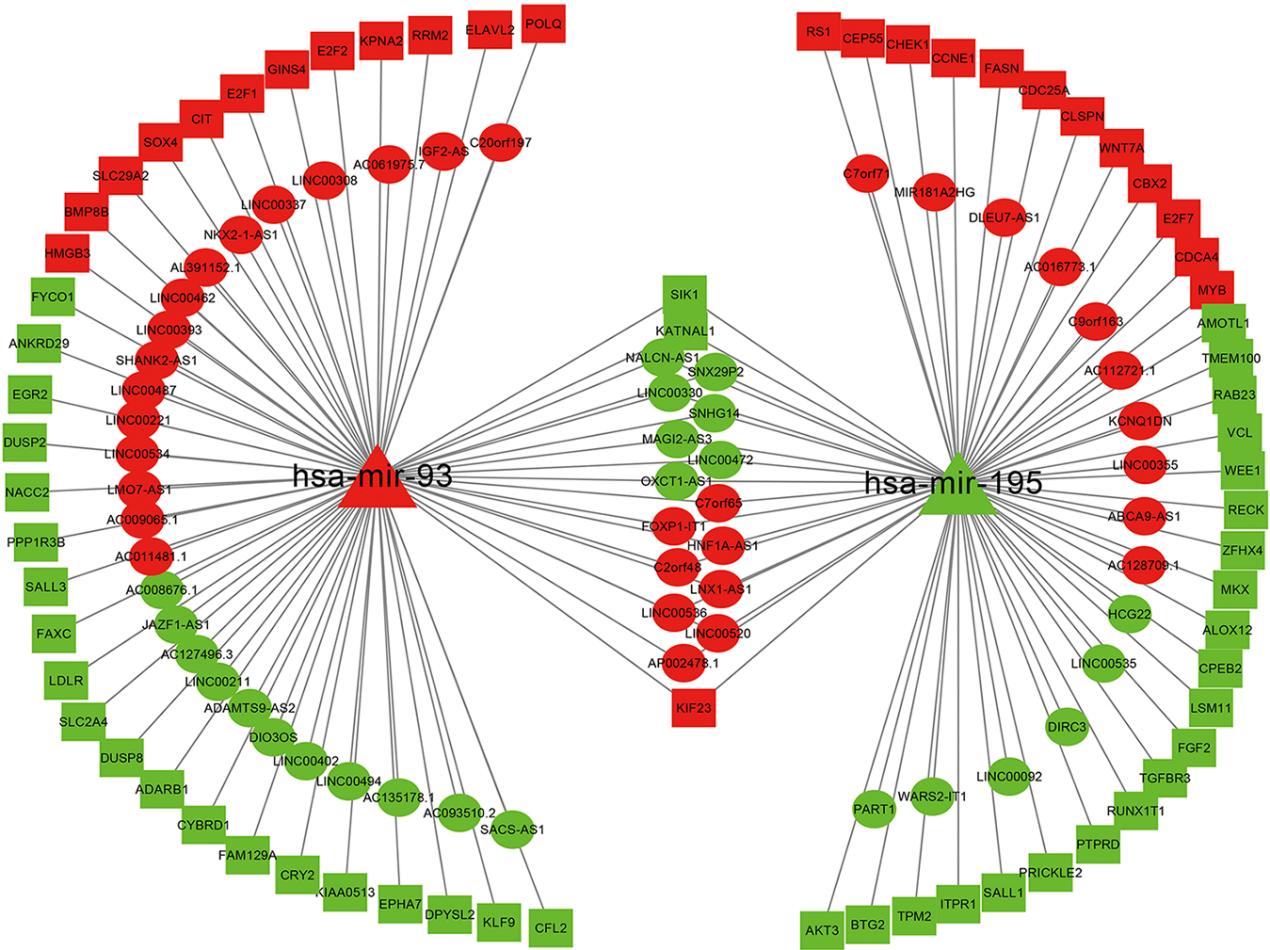
Network related to miRNA-195 and miRNA-93 in bladder cancer. Ellipse represents for DElncRNAs, triangle represents for DEmiRNAs, and rectangle represents for DEmRNAs. Red denotes upregulations; green denotes downregulations.

We extracted two key miRNAs and their linked mRNAs and lncRNAs from a three-layer network. Then rebuilt a new subnetwork, which included 71 mRNAs, 2 miRNAs, 73 lncRNAs, and 144 edges. The subnetwork was visualized with Cytoscape software and was shown in **Figure 4**.

**Figure 5:**
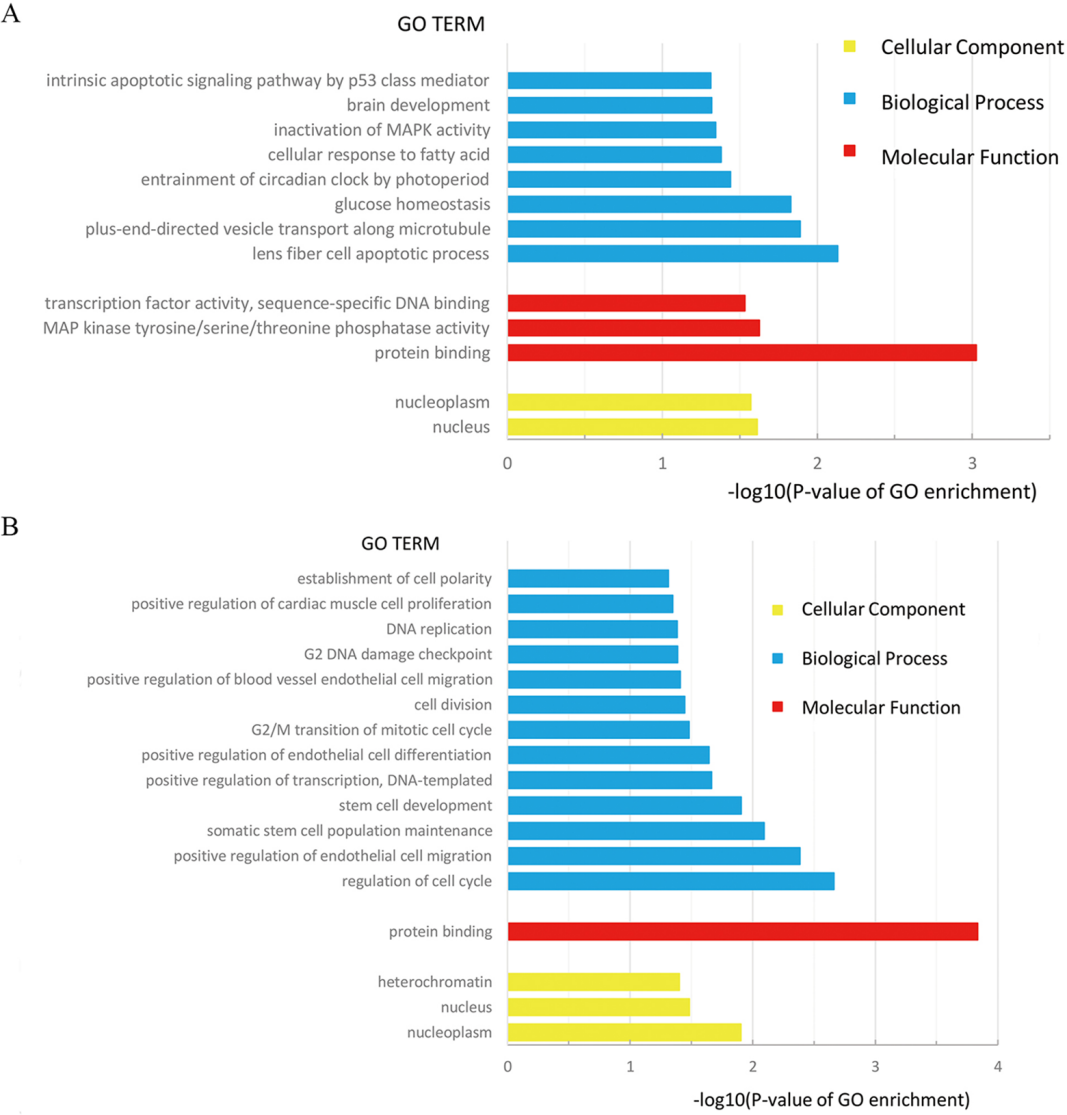
**(A)** GO Term of 35 target genes of miRNA-93; **(B)** GO Term of 36 target genes of miRNA-195.

Through GO analysis of 35 target genes of miRNA-93 (**Figure 5A**), we found that the dysregulated mRNAs associated with several -BP-, including lens fiber cell apoptotic process and plus-end-directed vesicle transport along microtubule. And they are also related to nucleus and nucleoplasm in -CC-. Furthermore, based on -MF- analysis, we conclude that miRNA-93 may be involved in the biological processes of BC by regulating the expression of its target genes. The functions of its target gene are mainly glucose homeostasis, protein binding and MAP kinase tyrosine/serine/threonine phosphatase activity and transcription factor activity, sequence-specific DNA binding. Among them, the discovery of MAP kinase serine phosphatase activity in molecular function further confirms the previously reported results that miRNA-93 promotes BC cell proliferation and invasion by targeting PEDF, a member of the serine protease inhibitor superfamily (Jiang et al., 2019). The details of the GO term associated with 35 DEmRNAs are shown in **Table 2**.

**Table 2:** Gene ontology (GO) analysis of 35 target genes of miRNA-93 (list by PValue).

Type	Term	Count	PValue	Genes
Molecular Function	protein binding	26	0.000942	E2F1, KIF23, E2F2, ADARB1, NACC2, HMGB3, EGR2, LDLR, GINS4, SOX4, ELAVL2, DPYSL2, EPHA7, DUSP2, CRY2, SLC2A4, CFL2, RRM2, CYBRD1, FAM129A, POLQ, KATNAL1, SIK1, CIT, FYCO1, KPNA2
Biological Process	lens fiber cell apoptotic process	2	0.007364701	E2F1, E2F2
Biological Process	plus-end-directed vesicle transport along microtubule	2	0.012853753	KIF23, FYCO1
Biological Process	glucose homeostasis	3	0.014869797	CRY2, SLC2A4, SOX4
Molecular Function	MAP kinase tyrosine/serine/threonine phosphatase activity	2	0.023620021	DUSP2, DUSP8
Cellular Component	nucleus	17	0.024386573	E2F1, KIF23, NACC2, ADARB1, HMGB3, EGR2, KLF9, GINS4, SOX4, SALL3, DUSP2, CRY2, RRM2, SIK1, KATNAL1, DUSP8, KPNA2
Cellular Component	nucleoplasm	11	0.026806886	KIF23, E2F1, E2F2, ADARB1, KLF9, RRM2, GINS4, SOX4, ELAVL2, POLQ, KPNA2
Molecular Function	transcription factor activity, sequence-specific DNA binding	6	0.029303045	SALL3, E2F1, E2F2, EGR2, KLF9, SOX4
Biological Process	entrainment of circadian clock by photoperiod	2	0.036302096	CRY2, SIK1
Biological Process	cellular response to fatty acid	2	0.041636205	E2F1, LDLR
Biological Process	inactivation of MAPK activity	2	0.045176392	DUSP2, DUSP8
Biological Process	brain development	3	0.047788991	EPHA7, EGR2, DPYSL2
Biological Process	intrinsic apoptotic signaling pathway by p53 class mediator	2	0.048703923	E2F1, E2F2

We also performed GO analysis on 36 target genes of miRNA-195 (**Figure 5B**), and functional enrichment results showed that these differentially expressed genes mainly (30/36) focused on protein binding in molecular function. At the same time, we found that the -CC- associated with mRNA is nucleoplasm (p = 0.012459971), and the -BP- most strongly associated with mRNA is the regulation of cell cycle (p = 0.002164798). In addition, the most relevant and only relevant -MF- associated with mRNA is the protein binding (P = 0.000145982). Based on these data, it can be speculated that the target genes of miRNA-195 can form complexes with different proteins to participate in regulating the cell cycle and positive regulation of endothelial cell migration, somatic stem cell population maintenance, stem cell development, accordingly, have certain effects on the occurrence and development of BC. The details of the GO term associated with 36 DEmRNAs are displayed in **Table 3**.

**Table 3:** Gene ontology (GO) analysis of 36 target genes of miRNA-195 (list by PValue).

Type	Term	Count	PValue	Genes
Molecular Function	protein binding	30	0.000145982	KIF23, CLSPN, E2F7, CBX2, CHEK1, CEP55, AMOTL1, VCL, CCNE1, RAB23, FASN, TMEM100, MYB, SIK1, FGF2, CDCA4, AKT3, RECK, PTPRD, RUNX1T1, CDC25A, ITPR1, WEE1, BTG2, SALL1, TGFBR3, LSM11, KATNAL1, WNT7A, ALOX12
Biological Process	regulation of cell cycle	4	0.002164798	CCNE1, FGF2, CDC25A, WEE1
Biological Process	Positive regulation of endothelial cell migration	3	0.004123996	WNT7A, FGF2, ALOX12
Biological Process	somatic stem cell population maintenance	3	0.008085217	SALL1, WNT7A, FGF2
Biological Process	stem cell development	2	0.012442813	WNT7A, FGF2
Cellular Component	nucleoplasm	12	0.012459971	KIF23, CLSPN, CCNE1, E2F7, SALL1, RUNX1T1, LSM11, CHEK1, CBX2, AKT3, CDC25A, WEE1
Biological Process	positive regulation of transcription, DNA-templated	5	0.021591723	CCNE1, SALL1, MYB, WNT7A, FGF2
Biological Process	positive regulation of endothelial cell differentiation	2	0.022696818	TMEM100, ALOX12
Cellular Component	nucleus	17	0.032803617	KIF23, CPEB2, E2F7, CHEK1, CBX2, WEE1, CDC25A, CCNE1, ZFHX4, SALL1, LSM11, MKX, SIK1, MYB, KATNAL1, FGF2, CDCA4
Biological Process	G2/M transition of mitotic cell cycle	3	0.032988025	CHEK1, CDC25A, WEE1
Biological Process	cell division	4	0.035939287	CCNE1, CDC25A, CDCA4, WEE1
Biological Process	positive regulation of blood vessel endothelial cell migration	2	0.038888456	AMOTL1, FGF2
Cellular Component	heterochromatin	2	0.039587501	SALL1, CBX2
Biological Process	G2 DNA damage checkpoint	2	0.040893995	CLSPN, CHEK1
Biological Process	DNA replication	3	0.041293616	CLSPN, CHEK1, CDC25A
Biological Process	positive regulation of cardiac muscle cell proliferation	2	0.044892884	TGFBR3, FGF2
Biological Process	establishment of cell polarity	2	0.048875575	WNT7A, WEE1

**Figure 6:**
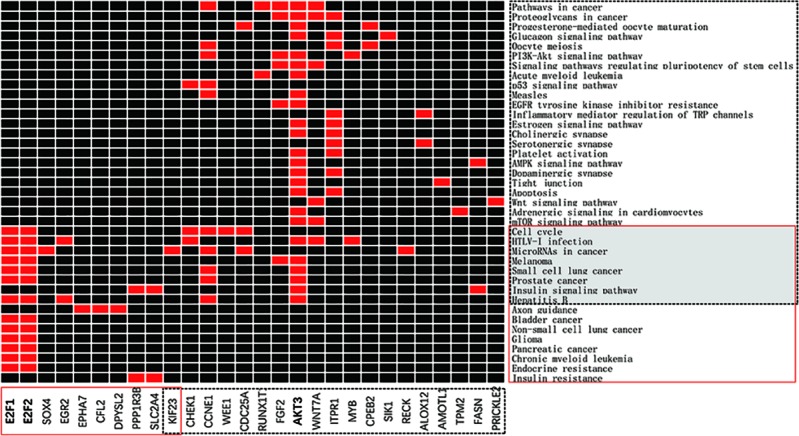
miRNA-targeted mRNA enrichment pathway. The x-axis represents the genes in the network, in which the red solid line is the target genes of miRNA-93, and the black dotted line is the target genes of miRNA-195. The y-axis represents the KEGG pathway, the red solid box is the enrichment pathway of the miRNA-93 target genes, and the black dotted box is the enrichment pathway of the miRNA-195 target genes.

KEGG enrichment analysis of metabolic pathways for miRNA-93 and miRNA-195 targeted mRNAs showed that a total of 28 genes are enriched in 39 pathways, of which 10 target genes of miRNA-93 are mainly involved in the regulation of 16 KEGG signaling pathways including cancer pathways and Hepatitis B and Axon guidance and other pathways, the 19 target genes of miRNA195 are mainly involved in the regulation of 31 KEGG signaling pathways including many cancer pathways and Cell cycle, HTLV-I infection (**Figure 6**). Demonstrating that miRNAs can play a role in a variety of BC signaling pathways. In addition, we found that the signaling pathways “MicroRNAs in cancer,” “HTLV-I infection,” and “cell cycle” are significantly enriched KEGG pathways, indicating that these three signaling pathways are closely related to BC. The details of the KEGG pathway enrichment are displayed in **Table 4**.

**Table 4:** KEGG pathways enriched by 71 DEmRNAs involved in the Hub network.

ID	Term description	Count	P-Value	Genes
mRNAs targeted by hsa-mir-93				
hsa05206	MicroRNAs in cancer	4	0.000143218	E2F1, KIF23, SOX4, E2F2
hsa05161	Hepatitis B	3	0.000308336	E2F1, E2F2, EGR2
hsa04360	Axon guidance	3	0.000526831	EPHA7, CFL2, DPYSL2
hsa05219	Bladder cancer	2	0.000663363	E2F1, E2F2
hsa05223	Nonsmall cell lung cancer	2	0.001204323	E2F1, E2F2
hsa05166	HTLV-I infection	3	0.001580128	E2F1, E2F2, EGR2
hsa05214	Glioma	2	0.00160289	E2F1, E2F2
hsa05212	Pancreatic cancer	2	0.001650553	E2F1, E2F2
hsa05218	Melanoma	2	0.00189891	E2F1, E2F2
hsa05220	Chronic myeloid leukemia	2	0.002002919	E2F1, E2F2
hsa05222	Small cell lung cancer	2	0.002743232	E2F1, E2F2
hsa05215	Prostate cancer	2	0.002929728	E2F1, E2F2
hsa01522	Endocrine resistance	2	0.003455352	E2F1, E2F2
hsa04931	Insulin resistance	2	0.00431996	PPP1R3B, SLC2A4
hsa04110	Cell cycle	2	0.005526668	E2F1, E2F2
hsa04910	Insulin signaling pathway	2	0.006869918	PPP1R3B, SLC2A4
mRNAs targeted by hsa-mir-195				
hsa04110	Cell cycle	4	0.00000555	CHEK1, CCNE1, WEE1, CDC25A
hsa05200	Pathways in cancer	5	0.0000299	CCNE1, RUNX1T1, FGF2, AKT3, WNT7A
hsa05205	Proteoglycans in cancer	4	0.0000382	FGF2, AKT3, WNT7A, ITPR1
hsa05166	HTLV-I infection	4	0.000093	CHEK1, AKT3, WNT7A, MYB
hsa04914	Progesterone-mediated oocyte maturation	3	0.00010659	CDC25A, AKT3, CPEB2
hsa04922	Glucagon signaling pathway	3	0.000119603	AKT3, ITPR1, SIK1
hsa05206	MicroRNAs in cancer	4	0.000160144	CCNE1, CDC25A, KIF23, RECK
hsa04114	Oocyte meiosis	3	0.000204986	CCNE1, ITPR1, CPEB2
hsa04151	PI3K-Akt signaling pathway	4	0.000265539	CCNE1, FGF2, AKT3, MYB
hsa04550	Signaling pathways regulating pluripotency of stem cells	3	0.00030972	FGF2, AKT3, WNT7A
hsa05221	Acute myeloid leukemia	2	0.001317859	RUNX1T1, AKT3
hsa04115	p53 signaling pathway	2	0.001901017	CHEK1, CCNE1
hsa05218	Melanoma	2	0.00200813	FGF2, AKT3
hsa01521	EGFR tyrosine kinase inhibitor resistance	2	0.002585615	FGF2, AKT3
hsa05222	Small cell lung cancer	2	0.002900294	CCNE1, AKT3
hsa05215	Prostate cancer	2	0.003097313	CCNE1, AKT3
hsa04750	Inflammatory mediator regulation of TRP channels	2	0.00372495	ITPR1, ALOX12
hsa04915	Estrogen signaling pathway	2	0.003798048	AKT3, ITPR1
hsa04725	Cholinergic synapse	2	0.004726968	AKT3, ITPR1
hsa04726	Serotonergic synapse	2	0.004808654	ITPR1, ALOX12
hsa04611	Platelet activation	2	0.005661192	AKT3, ITPR1
hsa04152	AMPK signaling pathway	2	0.005929498	AKT3, FASN
hsa04728	Dopaminergic synapse	2	0.006389399	AKT3, ITPR1
hsa05162	Measles	2	0.0069621	CCNE1, AKT3
hsa04530	Tight junction	2	0.007256895	AKT3, AMOTL1
hsa04910	Insulin signaling pathway	2	0.007256895	AKT3, FASN
hsa04210	Apoptosis	2	0.007356404	AKT3, ITPR1
hsa04310	WNT signaling pathway	2	0.007658643	WNT7A, PRICKLE2
hsa05161	Hepatitis B	2	0.00796643	CCNE1, AKT3
hsa04261	Adrenergic signaling in cardiomyocytes	2	0.008279735	AKT3, TPM2
hsa04150	mTOR signaling pathway	2	0.008814086	AKT3, WNT7A

Since E2F1 and E2F2 are enriched in various KEGG signaling pathways, they may be important target genes of miRNA-93. Similarly, AKT3 may be an important target gene of miRNA-195. From the functions annotated in the genes E2F1 and E2F2 in GO, we can speculate that the genes E2F1 and E2F2 are present on the nucleoplasm, and participate in the lens fiber cell apoptosis process or intrinsic apoptotic signaling pathway by p53 class mediator by forming a complex with a certain protein or transcription factor activity, sequence-specific DNA binding, which is consistent with the results reported previously (Castillo et al., 2015). In addition, PI3K-Akt and AMPK are reported to be positive and negative regulators of mTOR activity, respectively, and mTOR is a well-known sensor for extracellular nutrients and growth factors, which converges many key signals and is widely involved in cell cycle, cell proliferation, and cellular metabolic processes, and has important biological roles in regulating apoptosis, physiological, and pathological processes (Ciuffreda et al., 2010). The PI3K-Akt-mTOR signaling pathway has been shown to play a central role in several cellular functions, including survival, proliferation, adhesion, migration, differentiation, and metabolism (Gao et al., 2003). Activated AMPK inhibits mTOR and further inhibits protein synthesis, cell proliferation, cell cycle progression, and angiogenesis (Jalving et al., 2010).This study found that the expression of AKT3 is associated with these pathways, suggesting that AKT3 may participate in important processes such as cell proliferation and apoptosis through these pathways, ultimately affecting the biological behavior of tumor cells.

KEGG analysis of the downregulated target genes of miRNA-93 revealed that EPHA7, CFL2, and DPYSL2 are significantly enriched in axon guidance pathway (P = 0.000129528), and PPP1R3B and SLC2A4 are significantly attached to insulin resistance pathway (p = 0.001718367), we hypothesized that the activated axon guidance pathway and the insulin resistance pathway may be responsible for the downregulation of miRNA-93 in BC. KEGG analysis of the upregulated target genes of miRNA-195 revealed that CHEK1, CCNE1, and CDC25A are significantly enriched in the cell cycle pathway (p = 0.00000888), and CHEK1, MYB, and WNT7A are enriched in HTLV-I infection pathway (p = 0.000077), CCNE1, KIF23, and CDC25A enriches microRNAs in cancer (p = 0.000117168), CHEK1 and CCNE1 enriches p53 signaling pathway (p = 0.000241969). Therefore, we believe that “cell cycle, HTLV-I infection, MicroRNAs in cancer, p53 signaling pathway” may be related to downregulation of miRNA-195 in BC.

### Survival Analysis

We performed Kaplan-Meier survival analysis to predict overall survival (OS) of DERNAs in BC. As a result, we found that 253 of the 1457 differentially expressed lncRNAs were related to survival, 38 of the 247 differentially expressed miRNAs were associated with survival, and 625 of the 3047 differentially expressed mRNAs were related to survival (**Supplementary Material Tables S1–S3**). Similarly, Kaplan-Meier survival analysis was performed on miRNAs/lncRNAs and mRNAs in the three-layer converged network, and we found that 39 of the 197 lncRNAs were related to survival, 15 of the 65 miRNAs were related to survival, and 90 of the 367 mRNAs were related to survival (**Supplementary Material Tables S4–S6**).

**Figure 7:**
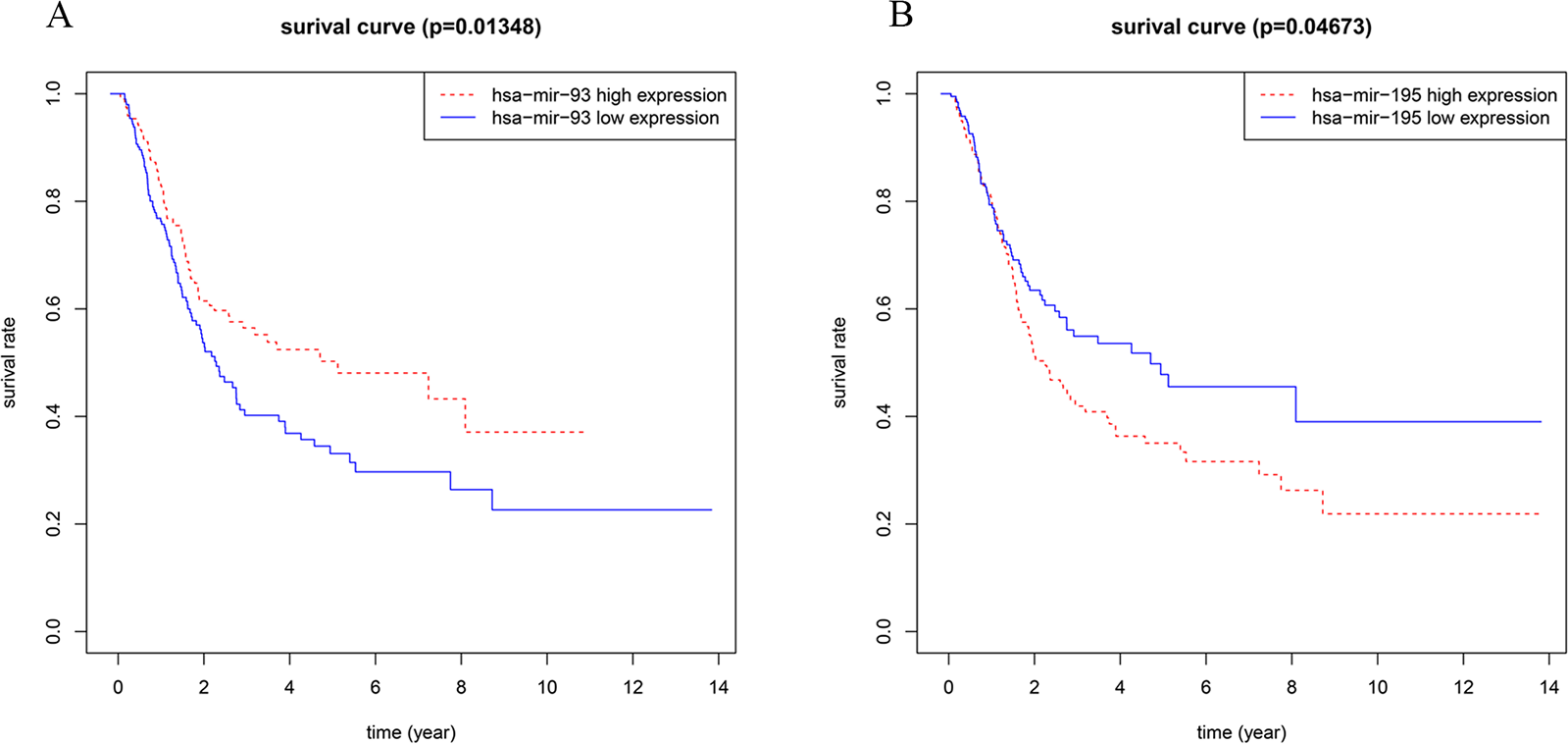
**(A)** Survival curves of OS for miRNA-93; **(B)** Survival curves of OS for miRNA-195. The x-axis denotes the time of OS and y-axis denotes the survival rate.

In addition, we also performed Kaplan-Meier survival analysis on miRNAs/lncRNAs and mRNAs in the subnetwork. Among them, miRNA-93 is highly correlated with overall survival of BC (**Figure 7A**). A large number of studies have shown that miRNA-93 can be widely involved in various biological behaviors of tumor cells and affect the development of tumors (Li et al., 2014). Therefore, in BC, the high expression of miRNA-93 may promote the proliferation of cancer cells and inhibit its apoptosis, which can lead to excessive proliferation and deterioration of cancer cells. miRNA-195 also has a correlation with the overall survival of BC (**Figure 7B**). Related studies (Fei et al., 2012) showed that miRNA-195 is a tumor suppressor gene, which inhibits cell proliferation and promotes apoptosis when normal expression, and downregulation leads to cancer, while miRNA -195 is downregulated in BC, so it may be closely related to the occurrence and development of BC. And we also found that nine lncRNAs (AC127496.3, ADAMTS9-AS2, C7orf65, LINC00536, MAGI2-AS3, SACS-AS1, AC016773.1, AC112721.1, and HCG22) were significantly associated with survival outcomes in patients with BC, among which MAGI2-AS3, C7orf65, and LINC00536 are lncRNAs linked to both miRNAs in the network. Twenty-four mRNAs (KATNAL1, ANKRD29, CFL2, DPYSL2, DUSP2, EGR2, FAM129A, KPNA2, LDLR, RRM2, AKT3, AMOTL1, BTG2, CPEB2, FASN, FGF2, LSM11, PTPRD, RAB23, RS1, RUNX1T1, TMEM100, VCL, and ZFHX4) are closely related to the survival prognosis of patients, 10 of which are target genes of miRNA-93, and 15 are target genes of miRNA-195. And KATNAL1 is a mRNA that is simultaneously regulated by two miRNAs in a network related to BC (**Supplementary Material Figure S1**).

## Discussion

A large number of studies have shown that the development of BC is highly correlated with the abnormal expression of noncoding RNA and protein-coding genes. However, there are still few studies on the key molecules of BC and abnormal gene expression patterns and pathways in BC. In this study, we constructed a three-layer network by merging lncRNA-miRNA regulatory network, miRNA-mRNA regulatory network, and lncRNA-mRNA coexpression network, and calculated the topology attributes of each node in the network, including degree, closeness centrality, and betweenness centrality. Our study suggested that miRNA-93 and miRNA-195 are controllers of the three-layer network associated with BC and a regulator of numerous target genes, and their dysregulation may be closely related to the pathogenesis of BC. A recent study also showed that miRNA-93 is upregulated in BC and is associated with tumor stage and lymph node metastasis (Jiang et al., 2019). And studies have reported that miRNA-195 inhibits glucose uptake and proliferation of BC T24 cells by inhibiting GLUT3 expression (Fei et al., 2012), another study found that miRNA-195 inhibits BC cell proliferation by inhibiting Cdc42 / STAT3 signaling (Zhao et al., 2015).

To clarify the significance of miRNA-93 and miRNA-195 in BC as accurately as possible, we extracted mRNAs and lncRNAs directly linked to miRNA-195 and miRNA-93 from a three-layer network, and built a new subnetwork. Then, we assigned miRNA-targeted mRNA to terms in GO and known pathways in the KEGG database. Through GO analysis, we found that DEmRNAs in the network is mainly concentrated in the protein binding in molecular function, suggesting that the protein-coding genes play vital roles in the development of BC. What’s more, the result from KEGG pathway analysis showed that dysregulated mRNAs contribute to the development of BC through abnormal activation of many pathways. Therefore, we concluded that miRNA-93 and miRNA-195 may participate in various biological processes by regulating the expression of target genes with different functions in BC, which may have an important impact on the occurrence and development of BC. In addition, in recent years, a large number of studies have shown that miRNA-93 and miRNA-195 play an important role in the development of tumors. miRNA-93 overexpression was associated with tumor progression, metastasis and poor prognosis in patients with head and neck squamous cell carcinoma (Li et al., 2015).And inhibiting the expression of miRNA-93 can inhibit the proliferation and migration of gastric cancer and liver cancer cells (Xu et al., 2012; Zhang et al., 2016). In addition, studies have shown that miR-195 has tumor suppressive properties in gastric cancer and glioma (Deng et al., 2013; Hui et al., 2013). Some evidences indicate that miRNA-195 can inhibit the ability of liver cancer cells to promote endothelial cell migration and angiogenesis, and can directly inhibit the migration and invasion of liver cancer cells, and inhibit liver cancer. Some evidences indicate that miRNA-195 can inhibit the ability of hepatoma cells to promote endothelial cell migration and angiogenesis, and can directly inhibit the migration and invasion of hepatoma cells, thereby inhibiting liver cancer (Wang et al., 2013).In osteosarcoma, miRNA-195 inhibits tumor migration and invasion by acting on fatty acid synthase (Mao et al., 2012).Based on these reports and current findings, we believe that the study of the mechanisms of action of miRNA-93 and miRNA-195 has profound implications for the clinical treatment of tumors.

Through survival analysis, we identified several RNAs associated with the prognosis of BC, some of which have not been reported. It is worth noting that the miRNAs of forming the hub nodes are related to survival. Previous studies have shown that the abnormal expression of miRNA-93 and miRNA-195 can promote the proliferation of cancer cells and inhibit their apoptosis (Fei et al., 2012; Li et al., 2014). Therefore, they may be involved in and influence the occurrence and development of BC. In addition, some of the genes in the subnetwork are often present in different cancers. For example, studies have found that ADAMTS9-AS2 is significantly downregulated in glioma tissues compared with normal tissues, and negatively correlated with prognosis (Yao et al., 2014). Downregulation of ADAMTS9-AS2 is associated with poor prognosis in patients with gastric cancer (Cao et al., 2018a). Low expression of ADAMTS9-AS2 is associated with overall survival in patients with ovarian cancer (Wang et al., 2018a). And ADAMTS9-AS2 is significantly upregulated in TSCC tissues of patients with lymph node metastasis and is closely related to poor prognosis (Li et al., 2019b). Besides, there are reports that the downregulation of MAGI2-AS3 in BC patients is associated with poor prognosis (Wang et al., 2018b) and the high expression of LINC00536 is negatively correlated with the survival rate of patients with BC(Li et al., 2019a), this is consistent with our results. These reports help identify and improve the accuracy of prognostic predictions.

In this work, we considered the relations of differentially expressed genes to construct the networks. As we know, the human body is a complex system. Maybe, there are some genes that do not differentially express have effects on the oncogenesis and progression of BC. In addition, we analyzed how the key genes affect other genes. It is meaningful to study the relation between key genes with proteins or other chemical molecules from the perspective of pathway function. We are currently working to integrate various valid data sets and try to develop more reliable prediction methods to provide reasonable and valuable candidate factors for the early diagnosis and treatment of BC.

## Conclusion

Taken together, we found that miRNA-93 and miRNA-195 are key factors for the development of BC by calculating the topology attributes of the nodes in the three-layer network. In addition, many mRNAs with different functions were found from KEGG and GO analysis, of which E2F1 and E2F2 are important target genes of miRNA-93, and AKT3 is an important target gene of miRNA-195, their dysregulation may be closely related to cell proliferation and apoptosis. Further the Kaplan-Meier curve analysis revealed that miRNA-93 and miRNA-195 are associated with poor prognosis of BC. In summary, our results have great significance in clinical practice, which can provide a reference for a better understanding of the occurrence and development of BC.

## Data Availability Statement

The datasets analyzed for this study can be found in the **Supplementary Material**.

## Author Contributions 

XW and YD designed the research ideas. XW collected and organized the data. XW, YD, JW, and YW analyzed the data. XW wrote the manuscript.

## Funding

This work was funded by The National Natural Science Foundation of China (grant numbers 21541006, 61772241).

## Conflict of Interest

The authors declare that the research was conducted in the absence of any commercial or financial relationships that could be construed as a potential conflict of interest.
