# Supplementary material for: Identification of the Key Factors Related to Bladder Cancer by lncRNA-miRNA-mRNA Three-Layer Network
Source: Front Genet. 2020 Jan 28;10:1398. doi: 10.3389/fgene.2019.01398 (PMC6997565; doi:10.3389/fgene.2019.01398)
Supplement: Supplementary file 1 [file DataSheet_1.pdf]

Table S1-All lncRNAs related to bladder cancer survival.

id	pvalue	id	pvalue	id	pvalue
ADAMTS9-AS1	2.00E-05	SATB2-AS1	0.00877	AC061975.1	0.02548
AP000894.2	2.00E-05	MKX-AS1	0.00887	AF015262.1	0.02568
AC104785.1	2.00E-05	LINC02416	0.00923	AC092171.4	0.02617
MAFG-DT	3.00E-05	LINC00603	0.00924	RNF144A-AS1	0.02624
LINC01776	5.00E-05	AC092376.2	0.00925	AC104071.1	0.02629
Z99289.1	8.00E-05	AC005150.1	0.00929	AC002398.2	0.02641
ADAMTS9-AS2	9.00E-05	LINC00351	0.0095	AL157400.2	0.02647
AP003500.1	0.00012	AC018809.1	0.00974	AC079313.2	0.02675
AC239584.1	0.00016	LINC01795	0.00996	AC002546.1	0.02686
AC005730.2	2.00E-04	AL136084.3	0.01043	AC012494.1	0.02688
GAS6-DT	0.00023	AP001189.3	0.011	ST8SIA6-AS1	0.02714
AL391704.1	0.00023	AC005863.1	0.01108	AL132655.2	0.02734
AL079343.1	0.00031	AP001189.1	0.01115	AC011510.1	0.0274
LINC01767	0.00041	AC090673.1	0.01149	AL139002.1	0.02748
AC108025.2	0.00041	CASC22	0.01167	AC092325.1	0.02751
AC021546.1	0.00044	AC105053.1	0.01205	STAM-AS1	0.02823
AC010331.1	0.00053	LINC02262	0.01213	AC015660.1	0.02853
WASIR2	0.00069	PSORS1C3	0.01226	AC090159.1	0.02866
MIR99AHG	0.00071	AC104126.1	0.01234	AC015712.2	0.02912
AC068196.1	0.00114	AF111167.2	0.01288	AL359979.1	0.02951
LINC00702	0.00115	AC120049.1	0.01301	AC009060.1	0.0297
AC090673.2	0.00116	LINC00683	0.01329	TARID	0.02971
MIR100HG	0.00125	AC025259.3	0.01343	MAGI2-AS3	0.0301
AP002884.1	0.00131	AC120498.2	0.01351	AC012603.1	0.03055
LINC01154	0.00137	LINC02522	0.01399	AC124276.2	0.03092
HCG22	0.00138	RORB-AS1	0.01431	AC004704.1	0.031
AC002454.1	0.00141	AL592429.2	0.01435	AC118755.2	0.03109

UBE2Q1-AS1	0.0016	AC079630.1	0.01438	AC097512.1	0.03116
LINC02434	0.00167	AC254629.1	0.01461	LEMD1-AS1	0.03169
LINC01197	0.00177	LINC01705	0.01471	AC092811.1	0.03183
LINC01647	0.00177	AL161431.1	0.01478	SMC2-AS1	0.03184
AC010643.1	0.00183	AC013457.1	0.0148	AP005230.1	0.03206
LINC01936	0.00201	AC093627.1	0.01491	MIR137HG	0.03218
AC022028.2	0.00207	AC116025.2	0.01539	G2E3-AS1	0.03273
AL355102.1	0.00211	AC103740.1	0.01568	LINC00698	0.03318
AC093390.1	0.00217	AC073174.1	0.01571	MIR9-3HG	0.03323
AP000864.1	0.00222	AC012085.2	0.01573	AP001628.2	0.03327
MEF2C-AS1	0.00247	AC092957.1	0.01577	AC022613.1	0.03328
AC053503.4	0.00258	AL161729.3	0.01592	BVES-AS1	0.03352
AL513218.1	0.00289	LINC02587	0.0162	AC116345.1	0.03357
PTPRD-AS1	0.00306	AC008708.2	0.01647	AC026333.4	0.03398
AL512785.1	0.00316	NCAM1-AS1	0.01678	AC126178.1	0.03442
AC002059.1	0.00326	AC017083.1	0.01682	AC112484.3	0.03462
LINC01833	0.00347	FLJ36000	0.01689	AC008403.3	0.03521
LINC02067	0.00358	AC017048.2	0.01691	LINC02584	0.03561
AC009093.4	0.00362	AL031665.1	0.01732	LINC00973	0.03591
CARMN	0.00365	AC092794.2	0.01735	LINC02074	0.03605
AC078778.1	0.00387	AC023908.3	0.0175	AC048337.1	0.037
LINC01179	0.00388	AC007495.1	0.0177	AC243773.1	0.03713
AC011298.1	0.00392	AC099066.2	0.01771	NKILA	0.03755
AL133255.1	0.00397	AC026369.1	0.01779	AF121898.1	0.0386
SIRLNT	0.00401	C7orf65	0.01817	AC044784.1	0.03879
AP003721.1	0.00436	AC026124.1	0.0182	AC005180.1	0.03906
AC079466.1	0.00446	AC093895.1	0.01863	NRIR	0.03907
AL590079.1	0.00459	SOX2-OT	0.01911	AC110285.6	0.0392
AL355488.1	0.00477	AC107959.1	0.01937	AC099850.3	0.03957

SACS-AS1	0.0048	AC005064.1	0.01948	DSCAS	0.04012
AL139130.1	0.00497	AC019211.1	0.01959	DNAH17-AS1	0.04022
AC025176.1	0.00498	LINC01352	0.01967	AC016705.2	0.04034
AL356270.1	0.0051	AC026691.1	0.01987	AC130456.1	0.04169
LINC00536	0.00518	AC017076.1	0.01989	AC018904.1	0.04189
AL355472.4	0.00519	AL390729.1	0.02052	LINC02195	0.04211
LINC01929	0.00526	AL136172.1	0.02058	AC105219.1	0.04232
ELDR	0.00534	AL355075.4	0.02059	AC016745.1	0.04253
AC016773.1	0.00544	LINC01605	0.02082	AC068473.4	0.04323
LINC00967	0.00544	AL356489.2	0.02097	AC092118.1	0.04399
AP001107.5	0.0055	AC004803.1	0.02103	AC108515.1	0.0443
AP000851.1	0.0058	AP000697.1	0.02177	AC025280.1	0.04446
AATBC	0.00666	LINC00460	0.02196	AC018716.1	0.04466
AL513329.1	0.00689	AC104123.1	0.02204	LINC01978	0.04491
TTTY16	0.007	AC009646.2	0.02206	AC013553.3	0.04562
AL122035.1	0.00701	AC092484.1	0.02271	AC011294.1	0.04577
AL589765.6	0.00704	LINC00879	0.02291	AC100858.3	0.04585
MYO16-AS1	0.00704	AC005746.3	0.02301	TRPM2-AS	0.04603
AC110491.1	0.00717	AL603832.1	0.02373	AC007773.1	0.04755
AC008063.2	0.00728	AC246817.1	0.02384	LINC02269	0.04757
LINC02167	0.00753	AC011511.3	0.02414	AC018521.6	0.04759
AL353801.3	0.00768	LINC02544	0.0244	AC124069.1	0.0477
AL078587.1	0.00773	AC116348.2	0.02455	AC127496.3	0.04777
AC112721.1	0.00808	AL139089.1	0.02476	AC025431.1	0.04792
AL158209.1	0.00817	AC034223.2	0.02479	AL161668.3	0.04854
SIRPG-AS1	0.00825	LNCAROD	0.02481	LINC02256	0.04859
LINC01385	0.00865	AC023824.1	0.02529	DLEU1-AS1	0.04862
AL024507.2	0.00868	TMEM132D-AS1	0.02545	LINC01952	0.04937
AC011921.1	0.00871				

Table S2-All miRNAs related to bladder cancer survival.

id	pvalue	id	pvalue	id	pvalue
hsa-mir-143	0.00707	hsa-mir-125b-2	3.00E-04	hsa-mir-3065	0.00122
hsa-mir-195	0.04673	hsa-mir-99a	0.00193	hsa-mir-4449	0.03327
hsa-mir-133b	0.00762	hsa-mir-6507	0.01993	hsa-mir-521-2	0.0182
hsa-mir-504	0.01008	hsa-mir-651	0.00251	hsa-mir-6715b	0.03837
hsa-let-7c	0.00293	hsa-mir-192	0.01846	hsa-mir-3189	0.02797
hsa-mir-145	0.01139	hsa-mir-33b	0.01816	hsa-mir-4636	0.02648
hsa-mir-548ba	0.01102	hsa-mir-490	0.0429	hsa-mir-944	0.00065
hsa-mir-93	0.01348	hsa-mir-4664	0.00369	hsa-mir-6728	0.00142
hsa-mir-425	0.03339	hsa-mir-3934	0.01955	hsa-mir-217	0.01663
hsa-mir-1258	0.04334	hsa-mir-3200	0.04676	hsa-mir-449b	0.03815
hsa-mir-7705	6.00E-05	hsa-mir-200b	0.04014	hsa-mir-216a	0.00022
hsa-mir-33a	0.00632	hsa-mir-200c	0.00302	hsa-mir-1224	0.04172
hsa-mir-125b-1	0.00035	hsa-mir-3913-1	0.01453		

Table S3-All mRNAs related to bladder cancer.

id	pvalue	id	pvalue	id	pvalue
HSPB6	1.00E-05	CLDN6	0.00664	MRGPRF	0.02219
EMP1	1.00E-05	E2F8	0.00665	KRT14	0.02223
TCHHL1	1.00E-05	PCDH7	0.00671	C6orf15	0.02236
KLK5	2.00E-05	SCEL	0.00688	RCAN1	0.02244
SHISAL1	3.00E-05	PHLDA1	0.00692	CC2D2A	0.02256
CRYAB	4.00E-05	ZDHHC15	0.00696	NR0B2	0.02282
BOC	6.00E-05	PRR11	0.007	SOX5	0.02307
LAMA2	7.00E-05	TSHZ3	0.00711	CLDN24	0.02308
PCARE	0.00011	FAT4	0.0072	MAMDC2	0.0236
PID1	0.00014	NRXN1	0.00723	ARID5B	0.02362
ABCC9	0.00015	CAMK2A	0.00724	FGFR1	0.02382

CASQ2	0.00017	CYP1B1	0.00738	HSPB2	0.02396
HSPG2	0.00017	TSPAN2	0.00745	FBXO41	0.02406
AOC3	0.00019	INHBA	0.00749	LRRK2	0.0242
OSBPL10	0.00019	PLEKHG4B	0.00752	MATN3	0.02463
GHR	0.00019	PLAC9	0.00763	FAM43A	0.02488
ADAMTSL3	2.00E-04	ADGRD1	0.0079	ZNF556	0.02497
SORCS1	0.00021	RASL12	0.00797	PREX2	0.02498
EPHB1	0.00022	SLC3A1	0.00798	SRPX	0.02502
MAP1B	0.00026	STON1	0.00799	NT5DC4	0.02511
MAP1A	0.00027	FBN2	0.00799	GAS7	0.02521
NCAM1	0.00027	DPF1	0.00802	HIST2H3D	0.02534
SCN4B	0.00028	NHSL2	0.00804	HRG	0.02547
HSPB7	3.00E-04	SPRR2F	0.00806	CNKSR2	0.02549
WDR97	0.00031	ITGB3	0.00812	DHCR7	0.0255
NEGR1	0.00033	JPH3	0.00819	PTGS1	0.0257
FLNC	0.00034	ATP2B4	0.00823	IGSF9B	0.02578
PDGFD	0.00034	ATP6V1B1	0.00834	EN1	0.02607
DIXDC1	0.00035	KRT38	0.00838	NTRK3	0.0262
GSN	0.00035	PLPPR4	0.0084	ZP4	0.02638
LDLR	0.00037	SGCB	0.00845	SRL	0.02639
SERPINB12	0.00039	C17orf47	0.00854	A1CF	0.02643
TNS1	0.00042	TCEAL2	0.00867	IGF2BP2	0.02648
SVIL	0.00042	MAOB	0.0087	ZFPM2	0.02653
NPTX1	0.00044	SAMD7	0.00878	VSTM2L	0.02667
OGN	0.00046	MYH2	0.0088	MFAP5	0.0268
RBMS3	0.00048	KCNS2	0.00884	PROZ	0.02682
S1PR1	0.00048	KLF17	0.0089	GYPC	0.02693
TMOD1	5.00E-04	AOX1	0.009	TUBA8	0.02702
PTGIS	0.00052	TUB	0.00913	KATNAL1	0.02703

CPED1	0.00053	ZBBX	0.00916	NTNG1	0.0271
DCHS1	0.00053	SLC6A17	0.00917	HCN3	0.02717
SYNC	0.00055	FAM129A	0.00927	GPC2	0.02726
MXRA7	0.00057	THSD4	0.00933	JAM2	0.02755
SCN7A	0.00057	MUC19	0.00933	KRTAP19-1	0.02765
KIAA1210	0.00058	WDR17	0.00937	PLN	0.02779
DOK6	0.00061	LSM11	0.0094	FZD7	0.0278
STRC	0.00061	RBM24	0.00948	FGF2	0.02815
ADAMTS12	0.00062	FAM124A	0.0095	NTF3	0.0282
CCDC80	0.00065	ANGPTL1	0.00974	FAM57B	0.02831
PCSK9	0.00066	GAD1	0.00978	DNAH11	0.02832
SETBP1	0.00068	ACTC1	0.00979	ZFP42	0.02852
SPON1	0.00069	F10	0.00981	ARMC4	0.02855
TENM2	7.00E-04	CFL2	0.00987	PDE4B	0.02861
ITGA7	0.00072	PCDHB8	0.00996	EPHB2	0.02861
POU5F1	0.00073	CAVIN2	0.01009	OLFM4	0.02871
SLC24A3	0.00076	ABI3BP	0.01011	FBLN5	0.02914
ADCYAP1R1	8.00E-04	TTLL7	0.01013	MGLL	0.02919
HYAL4	0.00082	ZMAT4	0.01013	PAQR4	0.02922
TNFAIP8L3	0.00083	EGR3	0.0103	KCNN3	0.02934
TMPRSS6	0.00083	DAAM2	0.01035	TBX5	0.02943
MFAP4	0.00085	NFATC1	0.01035	BUB1B	0.0296
GULP1	0.00086	PRSS56	0.01035	OTOG	0.02977
TCEAL5	0.00086	MTHFD1L	0.01038	BAG2	0.03014
TGFB1I1	0.00087	ZPLD1	0.01039	CAMKV	0.03048
TCHH	9.00E-04	PDE1C	0.0104	C7	0.03084
ECM1	0.00095	BDNF	0.01042	LMOD1	0.03089
RASSF3	0.00096	GALNT15	0.01052	OR2I1P	0.03092
ITIH2	0.00096	CLEC12B	0.01052	TRIM71	0.03096

MSRB3	0.001	SNED1	0.01054	PCDH20	0.03118
PRKG1	0.00103	OLFML3	0.01058	TRPC3	0.03131
RHOJ	0.00103	STRA8	0.01062	PDZRN3	0.03132
YDJC	0.00106	CDON	0.01063	TRIML2	0.03145
HOXB5	0.00112	AMOTL1	0.01065	CST4	0.03155
PRPH	0.00118	DUSP2	0.0108	SYNM	0.03188
SYNPO2	0.00122	MAP3K20	0.01086	HS3ST3A1	0.03194
BHMT2	0.00129	CDKL1	0.01086	SOBP	0.03247
PRELP	0.00129	CACNA1I	0.01087	F2	0.03268
DGKB	0.00135	HES2	0.01104	ADCYAP1	0.03288
SPARCL1	0.0014	SYPL2	0.0111	RAMP1	0.03289
KCNB1	0.00141	PDE1A	0.01115	SCNN1G	0.03296
UNC5C	0.00143	PTPRD	0.01123	AKT3	0.03307
PLA2G2F	0.00143	TMEM255A	0.01129	SH2D6	0.03313
LRFN2	0.00146	LRRN4	0.01145	PCDH9	0.0333
RS1	0.00146	HGFAC	0.01149	MUC16	0.03336
CTSV	0.00151	NEXMIF	0.01153	EDNRA	0.0334
GALNT16	0.00153	DAPL1	0.01156	SLMAP	0.03343
FAM110B	0.00155	CPEB2	0.01157	DNAJB5	0.03343
RBBP8NL	0.00156	KCNQ5	0.01163	PTGFR	0.03354
INSYN1	0.00161	TRIB3	0.01164	TMPRSS11E	0.03372
REEP1	0.00165	SPEG	0.01167	CDKN3	0.03381
IGSF10	0.00169	ADRA1D	0.01172	FBN1	0.03395
DCHS2	0.00169	PDE5A	0.01188	ALPG	0.03407
VSTM4	0.0017	RAB23	0.01191	COLEC12	0.03411
JAM3	0.00171	TXLNB	0.01194	GPIHBP1	0.03413
GEM	0.00175	RPTN	0.01202	ANXA6	0.03426
C2orf40	0.00178	CSRP1	0.01221	ADCY5	0.0343
COL14A1	0.00178	SCG2	0.01224	ZIC2	0.03436

CACNA2D1	0.00179	MYL3	0.01234	VCL	0.03443
NFIA	0.00181	MYH11	0.01243	ASXL3	0.03446
NEURL3	0.00182	ZFHX4	0.01244	DMD	0.03485
MEOX2	0.00188	GPR135	0.01261	SHISA3	0.03495
MEIOB	0.0019	FAM163B	0.01262	TMEM45A	0.03502
LHFPL6	0.00194	SPANXD	0.01265	TTC23L	0.03504
CRTAC1	0.00194	LATS2	0.01279	MCAM	0.03518
MYC	0.00195	SLC39A5	0.01287	MLIP	0.0355
HAVCR1	0.00204	TAGLN	0.01291	KRT4	0.03559
AIFM3	0.00204	SCN3B	0.01292	SORBS1	0.03566
ENKUR	0.00208	RGS22	0.01296	SMPDL3B	0.03569
PTGER3	0.00215	EPGN	0.01309	JAZF1	0.0357
CSPG4	0.00221	OTOF	0.0131	FGFBP1	0.03572
PTPRR	0.00221	CAP2	0.01321	CDC20	0.03573
FGF10	0.00226	BOLA2B	0.01358	FASN	0.03576
VSIG1	0.00233	RYR2	0.01369	PPM1N	0.03599
TM4SF19	0.00242	GNG11	0.01371	CNRIP1	0.03614
PRSS35	0.00243	CKMT2	0.01388	LBX2	0.03617
SH3GL3	0.00243	RUNX1T1	0.01388	ITGA1	0.0362
RAD54B	0.00246	TMPRSS11F	0.01388	FBXL7	0.03623
CALD1	0.00247	EPS8L3	0.01389	PM20D1	0.03623
TMEM132A	0.00249	EDNRB	0.01399	MYH7B	0.03624
BCHE	0.00252	PSD2	0.01401	KIF12	0.03637
KANK4	0.00263	NRXN2	0.01423	STARD9	0.0366
SERPINB2	0.00268	SLC23A3	0.01434	CLEC3B	0.0368
PDLIM3	0.00282	ARID3C	0.01447	LCN12	0.03714
FHL1	0.00285	CYP4F8	0.0146	MRVI1	0.03731
ZGRF1	0.00287	PRSS41	0.01467	UGT2A3	0.03746
GARNL3	0.00289	SHISA6	0.01468	HDAC4	0.03775

TPM1	0.0029	CPEB1	0.0147	RLN2	0.03782
SLIT2	0.00294	FGF11	0.01475	SSC4D	0.03789
MYLK	0.00295	CTGF	0.0149	MSH5	0.03799
CPXM2	0.00297	EPHA6	0.01512	OMD	0.03813
TOX3	0.00297	C12orf75	0.01514	ACTA2	0.03822
SGCD	0.00298	SCRG1	0.01536	PITX1	0.03859
PBOV1	0.003	RSPO3	0.01539	RRM2	0.0386
DPYSL2	0.00301	FSTL5	0.0154	SIM2	0.03871
FAM180A	0.00304	MAGEE2	0.01544	JUN	0.03898
SAPCD1	0.00306	TMEM229A	0.01562	SMC1B	0.03905
ADCY2	0.00312	LCE1F	0.01565	CYP2C9	0.03915
RHBG	0.00312	DPYSL3	0.01574	CRISPLD2	0.03944
COMP	0.0032	CYP3A7	0.01576	FOXP2	0.03962
SPAG4	0.00331	PLXNA4	0.01577	FILIP1	0.03983
NUDT10	0.00334	PTHLH	0.01583	SLC23A1	0.04005
MEPE	0.00339	ADRB3	0.01597	NAV3	0.04011
PDGFRA	0.00346	CDH19	0.01612	CYS1	0.04027
SLC2A6	0.0035	ABCA4	0.01612	TRIM63	0.04056
RGS2	0.00354	GPAT3	0.01613	METTL24	0.04059
SCN2B	0.00355	PODNL1	0.01618	ZEB1	0.0408
PCDH18	0.00363	CXCL12	0.01623	TMEM191C	0.0409
PDZD3	0.00363	PPARGC1A	0.01636	PRSS50	0.04169
CYTL1	0.00365	BTG2	0.01648	TPSD1	0.0422
MUC22	0.00367	SRD5A2	0.01662	KCND3	0.04224
PPFIA2	0.00374	CLIC4	0.01668	TMEM108	0.04241
LDB2	0.00379	CSRNP3	0.0167	ABCA8	0.04245
PALLD	0.00382	PSAPL1	0.0167	C1QL4	0.04262
LIME1	0.00383	CSDC2	0.01672	ANKRD29	0.04269
WWTR1	0.00384	NKX6-2	0.01674	LBX1	0.04271

ADRA2A	0.00384	HIST1H4C	0.01674	SLC30A3	0.04278
ZNF432	0.00384	SEMA3A	0.01693	RERG	0.04287
CNTN1	0.00394	TMEM35A	0.01707	CST1	0.04288
FXYD6	0.00404	KCNJ8	0.01731	DES	0.04297
P3H4	0.00406	MAPRE2	0.01738	PKHD1L1	0.04304
TRPM2	0.00429	PENK	0.01751	SAPCD2	0.04305
SHD	0.0043	EML1	0.01756	CHAT	0.04308
CTSG	0.00432	IGFBP5	0.01781	BVES	0.04375
SGK2	0.00435	PGR	0.0179	THBS1	0.04378
NRP2	0.00449	GUCA1C	0.0179	RSPO2	0.04382
C8orf88	0.00452	IGDCC4	0.01791	CRYBA2	0.04388
METTL11B	0.00457	TMEM132C	0.01792	CDCA2	0.04408
FBXO32	0.00461	AGTR1	0.01793	TRIML1	0.0441
CCIN	0.00461	NOVA1	0.01796	HDGFL1	0.04424
ADAMTS16	0.00462	ADAMTS9	0.01811	HAND2	0.04431
SLC5A11	0.00465	HEPH	0.01813	KCNE4	0.04454
MYADM	0.00489	TGFBR3L	0.01823	SUMO4	0.04458
IL1RAPL1	0.00498	FOXD1	0.01844	TRPC4	0.04462
HSD3B1	0.00503	OLFML1	0.01848	ROR2	0.04477
CHRDL1	0.00508	TNN	0.01866	GABRA2	0.04494
KCNMA1	0.00513	PRRG3	0.01868	TCEAL7	0.04517
FAIM2	0.00515	SLC22A3	0.0187	SLX1B	0.04565
TMEM100	0.00539	MALL	0.01878	EGR1	0.04568
SPTA1	0.0054	TFAP2B	0.01888	PEG3	0.04569
EBF1	0.00544	SFRP2	0.019	C4orf48	0.04617
SMOC2	0.00549	LRFN5	0.01905	GATA6	0.0463
DDR2	0.00556	S100A7A	0.01924	EPHA3	0.04658
PLSCR4	0.00559	ADGRA2	0.01939	UCN2	0.0466
CTSE	0.00559	AARD	0.0194	GPC6	0.04672

DSTN	0.00563	PCP2	0.01942	RSPO1	0.04674
OR7A5	0.00565	DTNA	0.01947	DMRT1	0.04696
FILIP1L	0.00569	PILRB	0.01954	GALNT17	0.04702
FHL5	0.00572	SCX	0.01962	DACT3	0.04736
FERMT2	0.00573	HSPA12A	0.01978	SPRR3	0.04752
PDZRN4	0.00583	CNN1	0.02002	SLC12A8	0.04773
EGR2	0.00585	HIST1H1C	0.02008	SLC18A3	0.04775
ARHGAP24	0.00592	SFRP1	0.02012	MMP9	0.04792
PLIN4	0.00592	COL21A1	0.02019	MYOCD	0.04816
NAP1L3	0.00595	DNASE1L2	0.0207	TRIB1	0.04836
NFE4	0.00603	SVEP1	0.02075	HMMR	0.04849
CCNB1	0.00606	PDE2A	0.02077	KLF4	0.04863
ANGPTL5	0.0061	FGF7	0.02097	STEAP1B	0.04875
BECN2	0.00612	PPEF1	0.02115	AKNAD1	0.04903
TBX1	0.00618	STXBP6	0.02121	CRHBP	0.04917
HIST1H3F	0.00625	KPNA2	0.02146	ARHGAP20	0.04918
NT5DC3	0.0063	A2M	0.02154	ADGRB3	0.04945
PMP2	0.00632	NFIL3	0.02169	ARHGEF25	0.04959
CDC42BPA	0.00635	TRIM55	0.02174	TPSAB1	0.04968
NAT8	0.00635	CMA1	0.02183	ACTL6B	0.04974
PLK1	0.00647	SAMD15	0.02186	RNFT2	0.04976
INSL6	0.00649	DCN	0.02192	HS3ST1	0.04993
C20orf204	0.00651	NXPH4	0.02205	CPA6	0.04999
AKAP6	0.0066				

Table S4-All lncRNAs related to bladder cancer survival in the three-layer network.

id	pvalue	id	pvalue	id	pvalue
ADAMTS9-AS1	2.00E-05	TTTY16	0.007	AC079313.2	0.02675

ADAMTS9-AS2	9.00E-05	MYO16-AS1	0.00704	AC012494.1	0.02688
AC108025.2	0.00041	AC110491.1	0.00717	AC011510.1	0.0274
WASIR2	0.00069	AC112721.1	0.00808	AL139002.1	0.02748
HCG22	0.00138	AC005150.1	0.00929	AC009060.1	0.0297
UBE2Q1-AS1	0.0016	LINC00351	0.0095	MAGI2-AS3	0.0301
AL355102.1	0.00211	PSORS1C3	0.01226	AC092811.1	0.03183
AC053503.4	0.00258	FLJ36000	0.01689	SMC2-AS1	0.03184
AC078778.1	0.00387	C7orf65	0.01817	MIR137HG	0.03218
SACS-AS1	0.0048	SOX2-OT	0.01911	AC005180.1	0.03906
LINC00536	0.00518	AC107959.1	0.01937	AC016745.1	0.04253
AC016773.1	0.00544	LINC00460	0.02196	AC127496.3	0.04777
TTTY16	0.007	AC002398.2	0.02641	AC025431.1	0.04792

Table S5-All miRNAs related to bladder cancer survival in the three-layer network.

id	pvalue	id	pvalue	id	pvalue
hsa-mir-216a	0.00022	hsa-mir-133b	0.00762	hsa-mir-192	0.01846
hsa-let-7c	0.00293	hsa-mir-145	0.01139	hsa-mir-425	0.03339
hsa-mir-200c	0.00302	hsa-mir-93	0.01348	hsa-mir-449b	0.03815
hsa-mir-33a	0.00632	hsa-mir-217	0.01663	hsa-mir-200b	0.04014
hsa-mir-143	0.00707	hsa-mir-33b	0.01816	hsa-mir-195	0.04673

Table S6-All mRNAs related to bladder cancer survival in the three-layer network.

id	pvalue	id	pvalue	id	pvalue
HSPB6	1.00E-05	CFL2	0.00987	FAM57B	0.02831
MAP1B	0.00026	DAAM2	0.01035	KCNN3	0.02934
NCAM1	0.00027	PRSS56	0.01035	CAMKV	0.03048
PDGFD	0.00034	BDNF	0.01042	LMOD1	0.03089

LDLR	0.00037	AMOTL1	0.01065	TRIM71	0.03096
NPTX1	0.00044	DUSP2	0.0108	TRPC3	0.03131
HOXB5	0.00112	PTPRD	0.01123	SYNM	0.03188
SYNPO2	0.00122	CPEB2	0.01157	AKT3	0.03307
RS1	0.00146	TRIB3	0.01164	SLMAP	0.03343
NFIA	0.00181	RAB23	0.01191	VCL	0.03443
MEOX2	0.00188	MYH11	0.01243	SORBS1	0.03566
AIFM3	0.00204	ZFHX4	0.01244	JAZF1	0.0357
FGF10	0.00226	LATS2	0.01279	FASN	0.03576
CALD1	0.00247	RUNX1T1	0.01388	FBXL7	0.03623
PBOV1	0.003	SHISA6	0.01468	MYH7B	0.03624
DPYSL2	0.00301	CTGF	0.0149	LCN12	0.03714
PDGFRA	0.00346	BTG2	0.01648	RRM2	0.0386
CHRDL1	0.00508	IGFBP5	0.01781	JUN	0.03898
TMEM100	0.00539	IGDCC4	0.01791	ZEB1	0.0408
EBF1	0.00544	FOXD1	0.01844	ANKRD29	0.04269
EGR2	0.00585	CNN1	0.02002	SLC30A3	0.04278
CCNB1	0.00606	KPNA2	0.02146	DES	0.04297
PMP2	0.00632	ARID5B	0.02362	THBS1	0.04378
E2F8	0.00665	LRRK2	0.0242	TCEAL7	0.04517
ITGB3	0.00812	IGF2BP2	0.02648	MMP9	0.04792
ATP2B4	0.00823	ZFPM2	0.02653	TRIB1	0.04836
FAM129A	0.00927	KATNAL1	0.02703	KLF4	0.04863
LSM11	0.0094	FZD7	0.0278	AKNAD1	0.04903
RBM24	0.00948	FGF2	0.02815	CRHBP	0.04917
ACTC1	0.00979	NTF3	0.0282	ACTL6B	0.04974

Figure S1-K-M curve analysis of survival-related mRNAs and lncRNAs in the subnetworks.

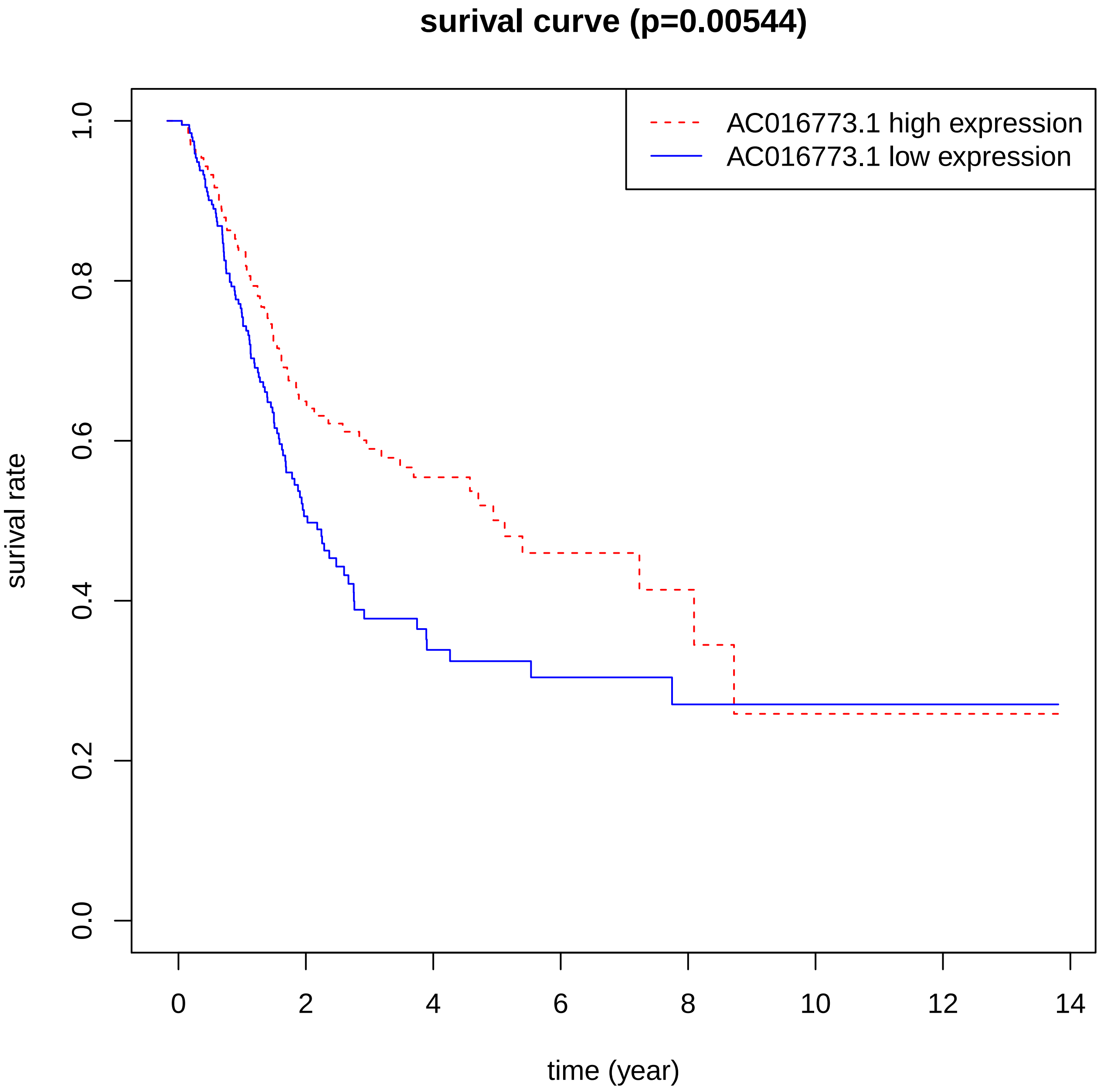
surival curve (p=0.00544)
AC016773.1 high expression
AC016773.1 low expression
surival rate
time (year)
0.0
0.2
0.4
0.6
0.8
1.0
0
2
4
6
8
10
12
14

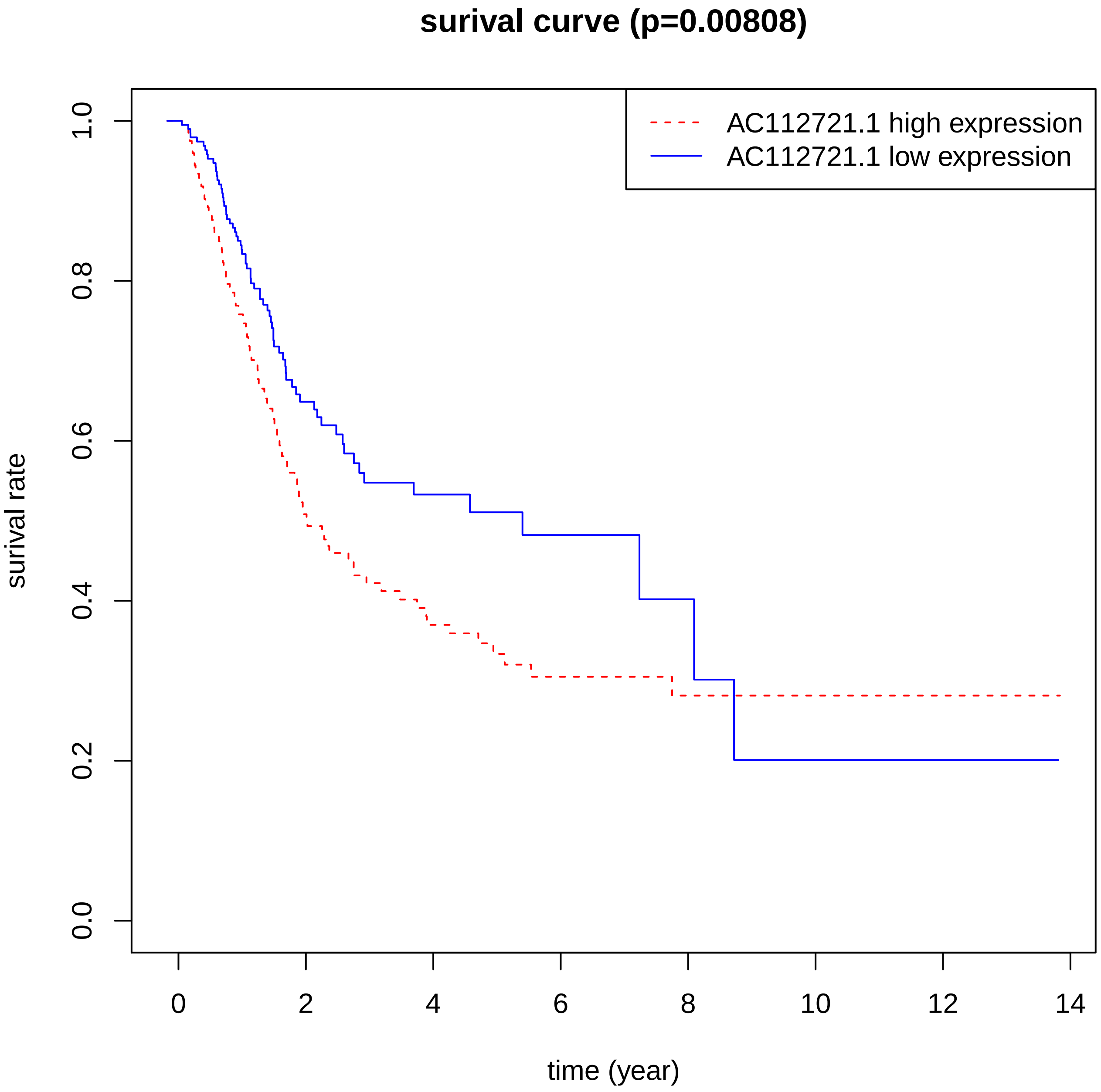

surival curve (p=0.00808)
AC112721.1 high expression
AC112721.1 low expression
surival rate
0.0
0.2
0.4
0.6
0.8
1.0
0
2
4
6
8
10
12
14
time (year)

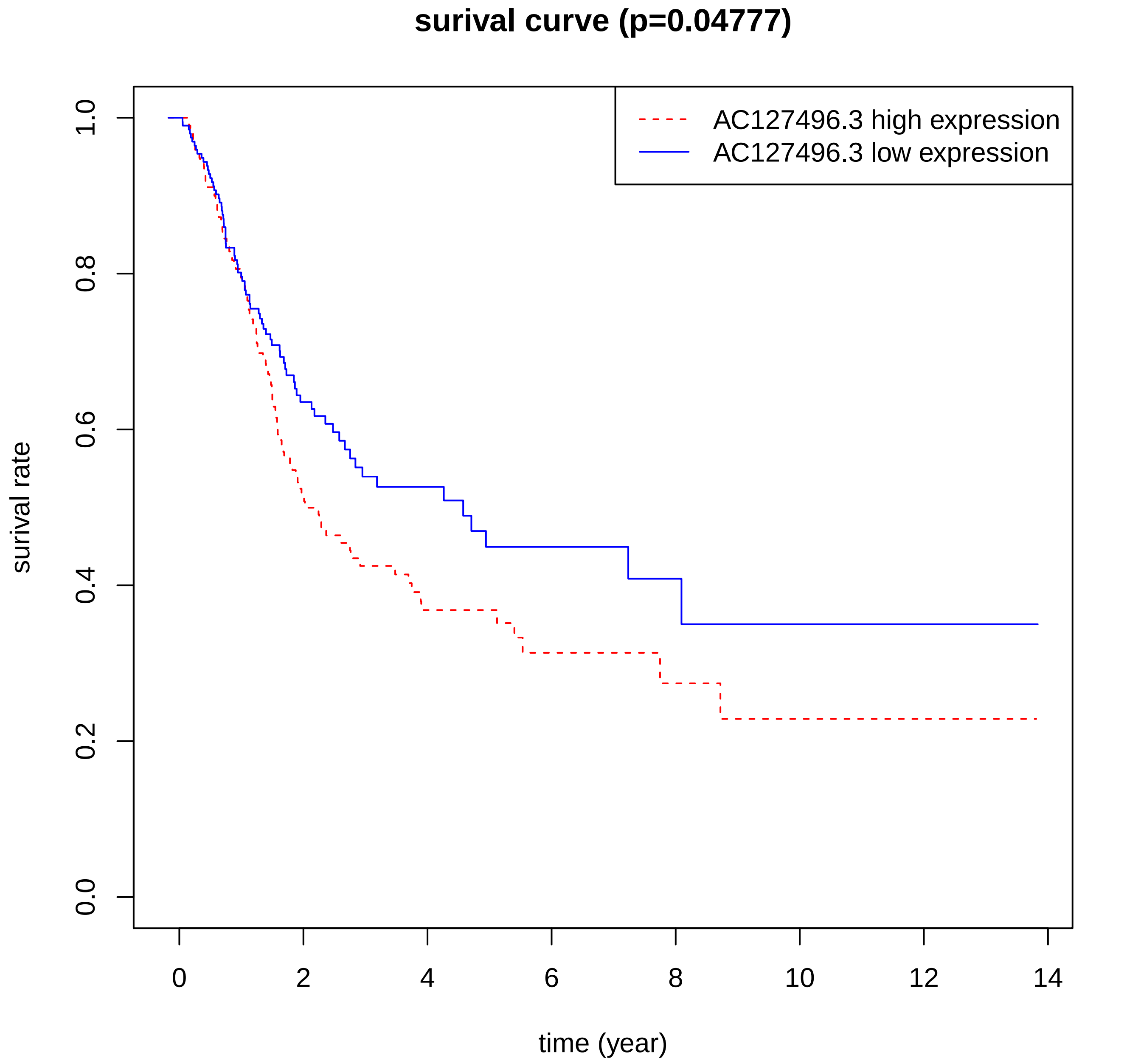

surival curve (p=0.04777)
AC127496.3 high expression
AC127496.3 low expression
surival rate
0.0
0.2
0.4
0.6
0.8
1.0
0
2
4
6
8
10
12
14
time (year)

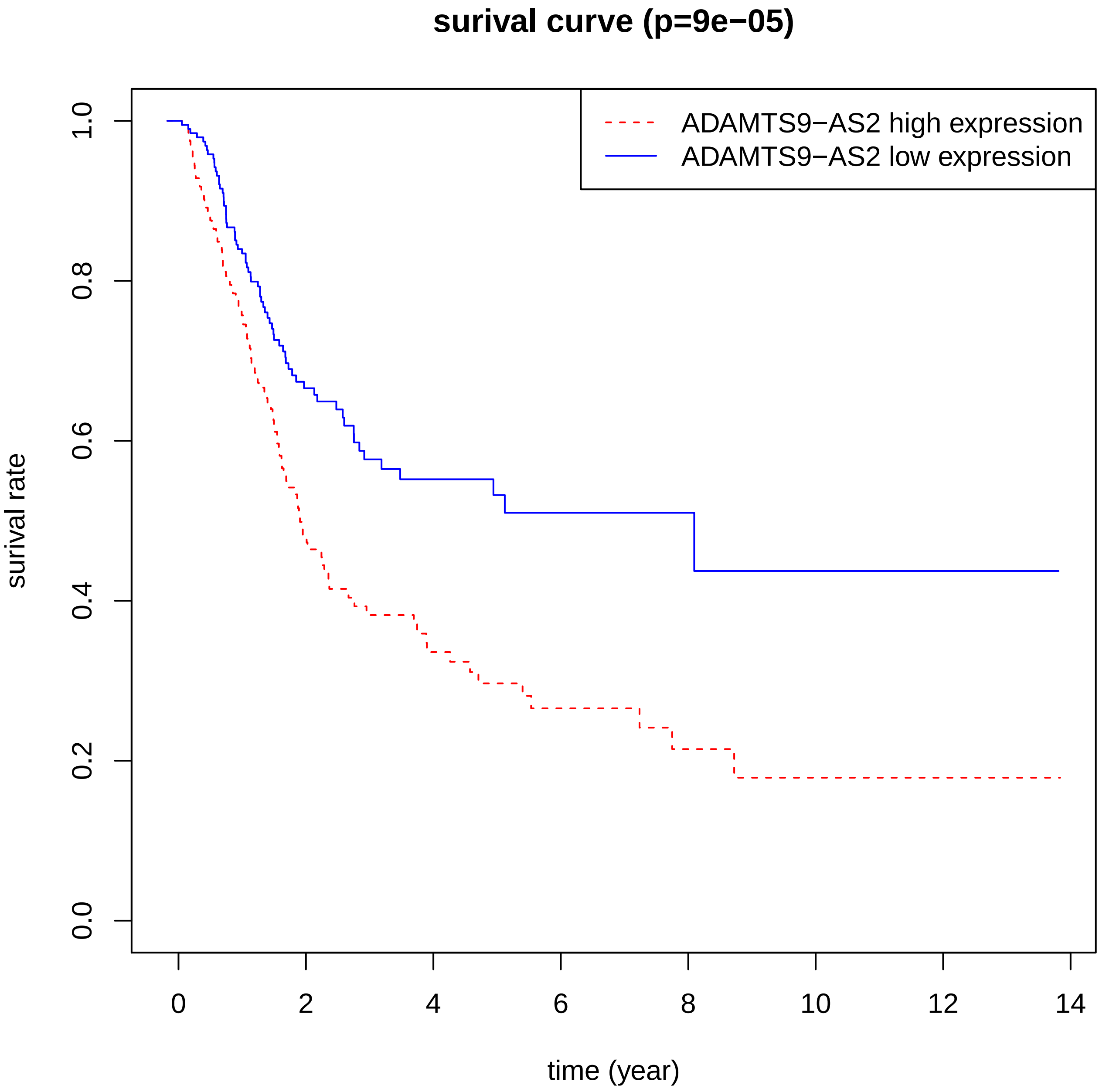
surival curve (p=9e−05)
ADAMTS9−AS2 high expression
ADAMTS9−AS2 low expression
surival rate
1.0
0.8
0.6
0.4
0.2
0.0
0
2
4
6
8
10
12
14
time (year)

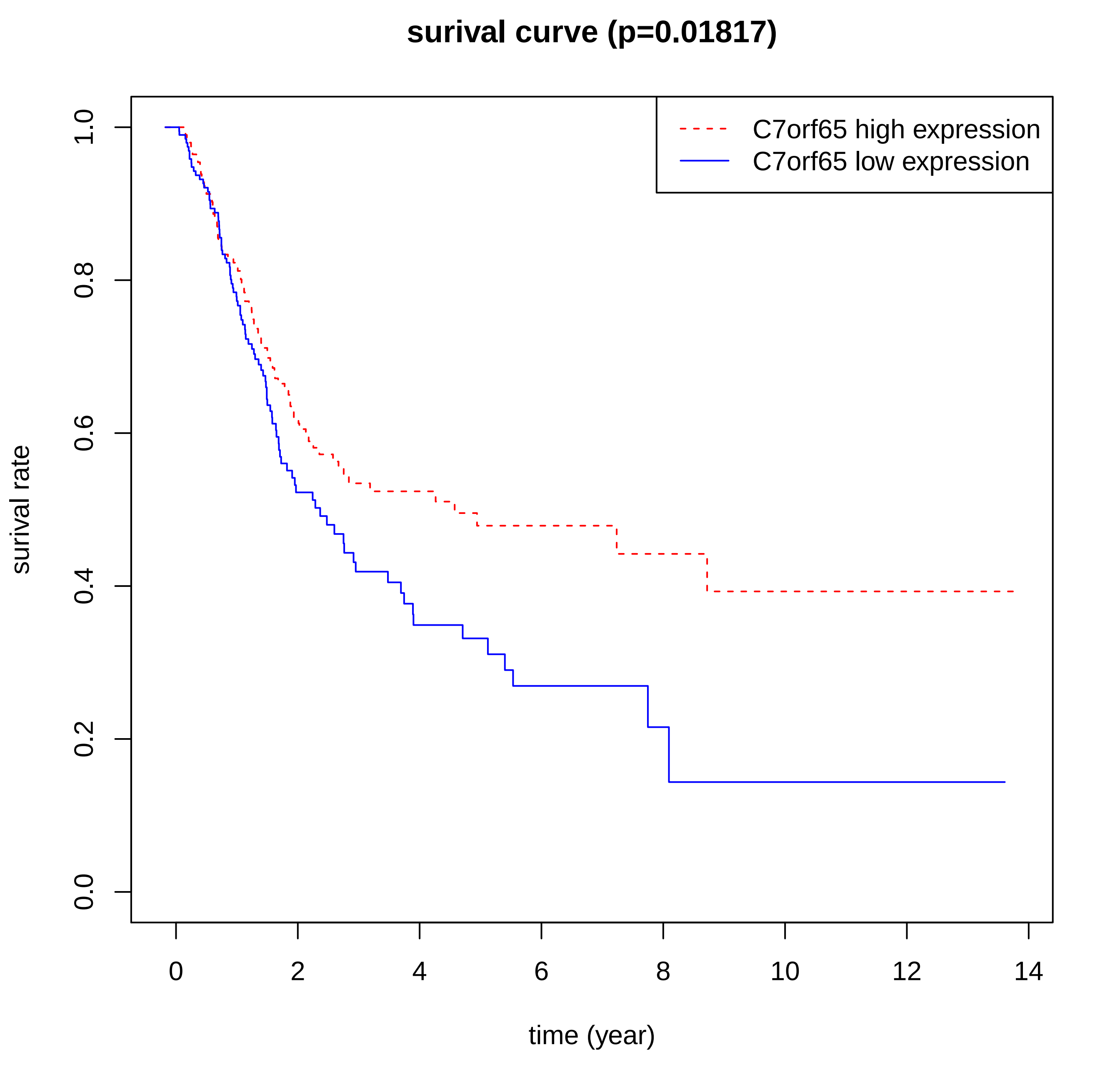
surival curve (p=0.01817)
C7orf65 high expression
C7orf65 low expression
surival rate
time (year)
0.0
0.2
0.4
0.6
0.8
1.0
0
2
4
6
8
10
12
14

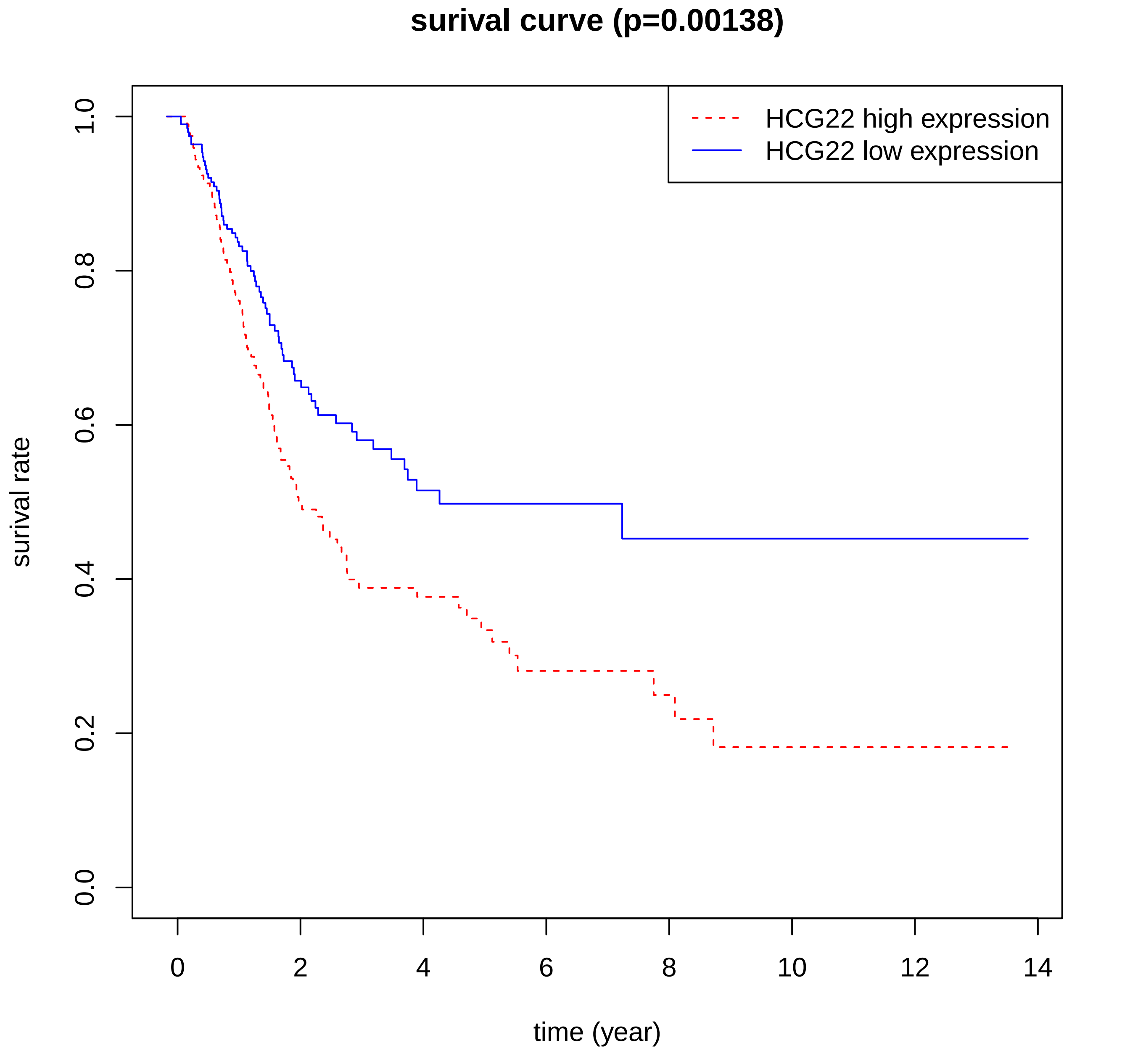

surival curve (p=0.00138)
HCG22 high expression
HCG22 low expression
surival rate
time (year)
0.0
0.2
0.4
0.6
0.8
1.0
0
2
4
6
8
10
12
14

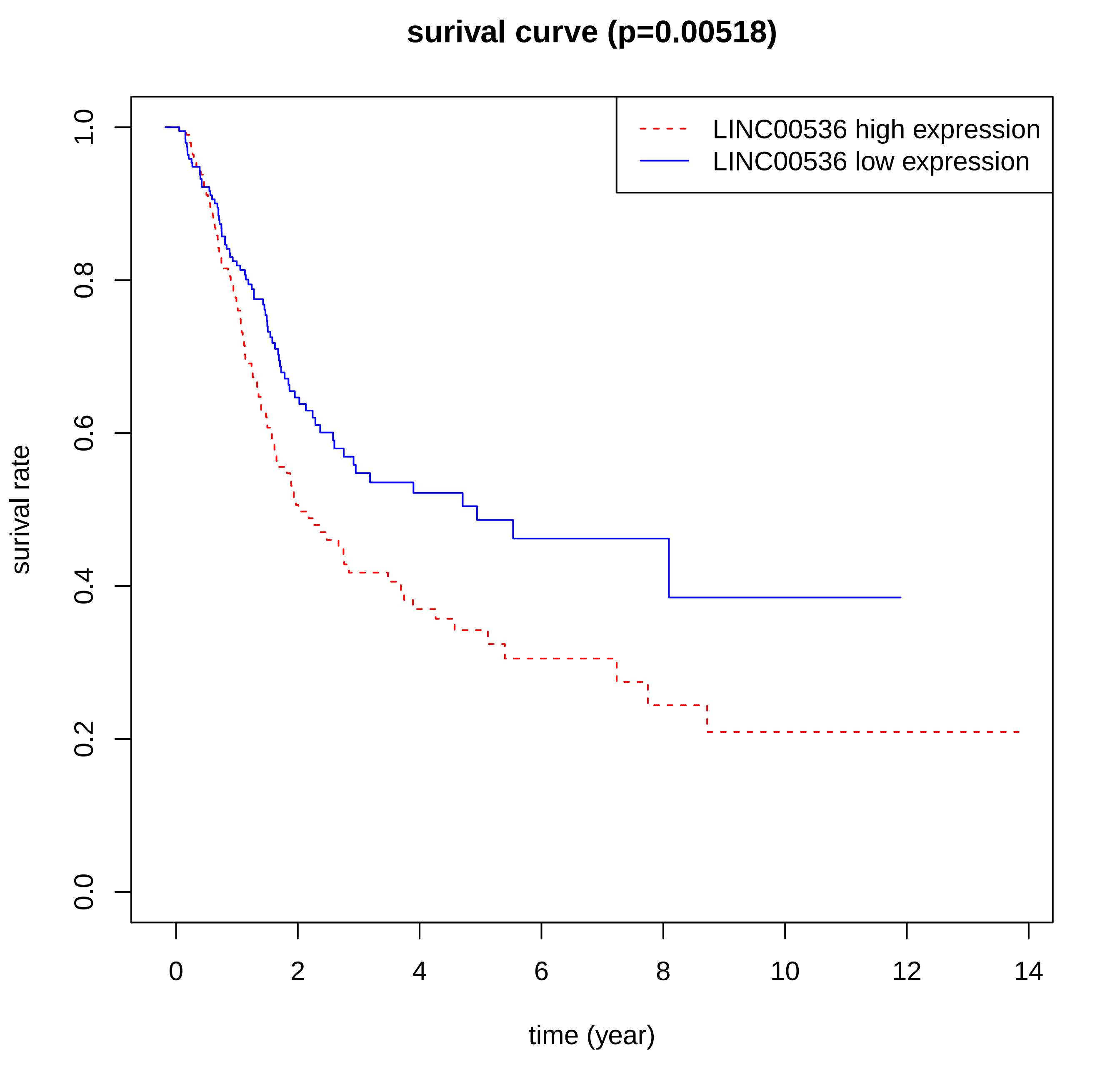
surival curve (p=0.00518)
LINC00536 high expression
LINC00536 low expression
surival rate
time (year)
0.0
0.2
0.4
0.6
0.8
1.0
0
2
4
6
8
10
12
14

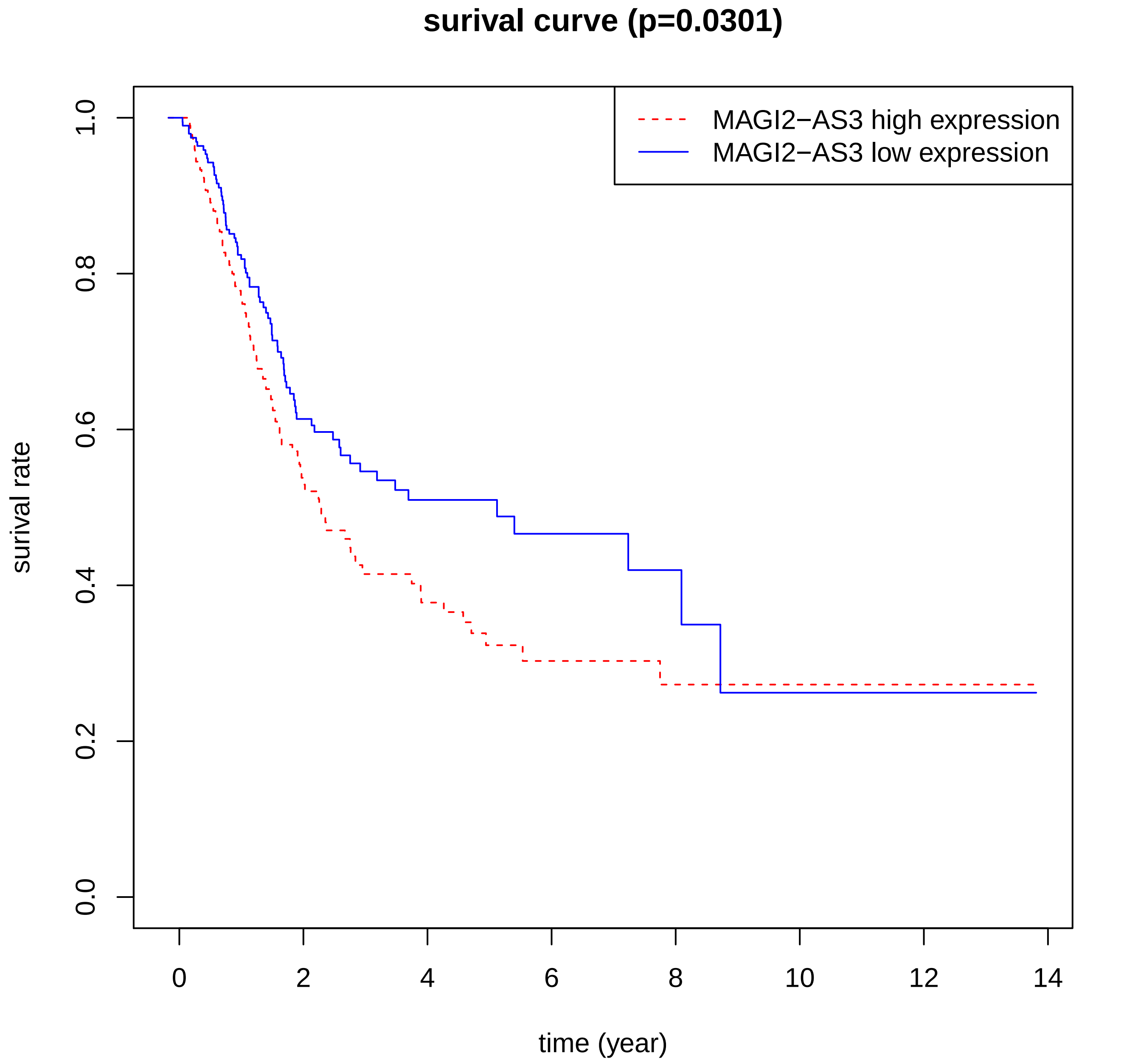
surival curve (p=0.0301)
MAGI2−AS3 high expression
MAGI2−AS3 low expression
surival rate
time (year)
0.0
0.2
0.4
0.6
0.8
1.0
0
2
4
6
8
10
12
14

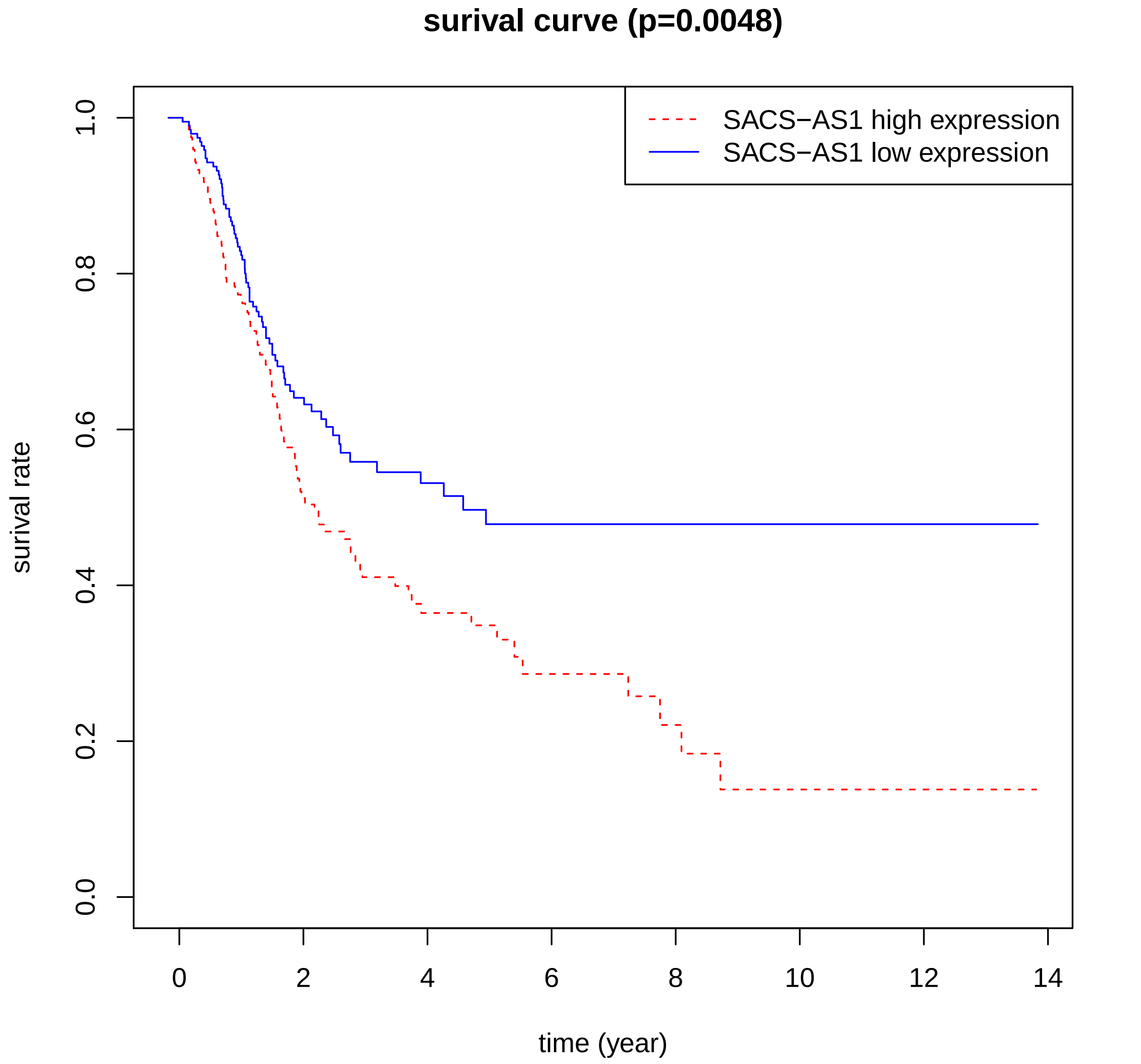
surival curve (p=0.0048)
SACS−AS1 high expression
SACS−AS1 low expression
surival rate
0.0
0.2
0.4
0.6
0.8
1.0
time (year)
0
2
4
6
8
10
12
14

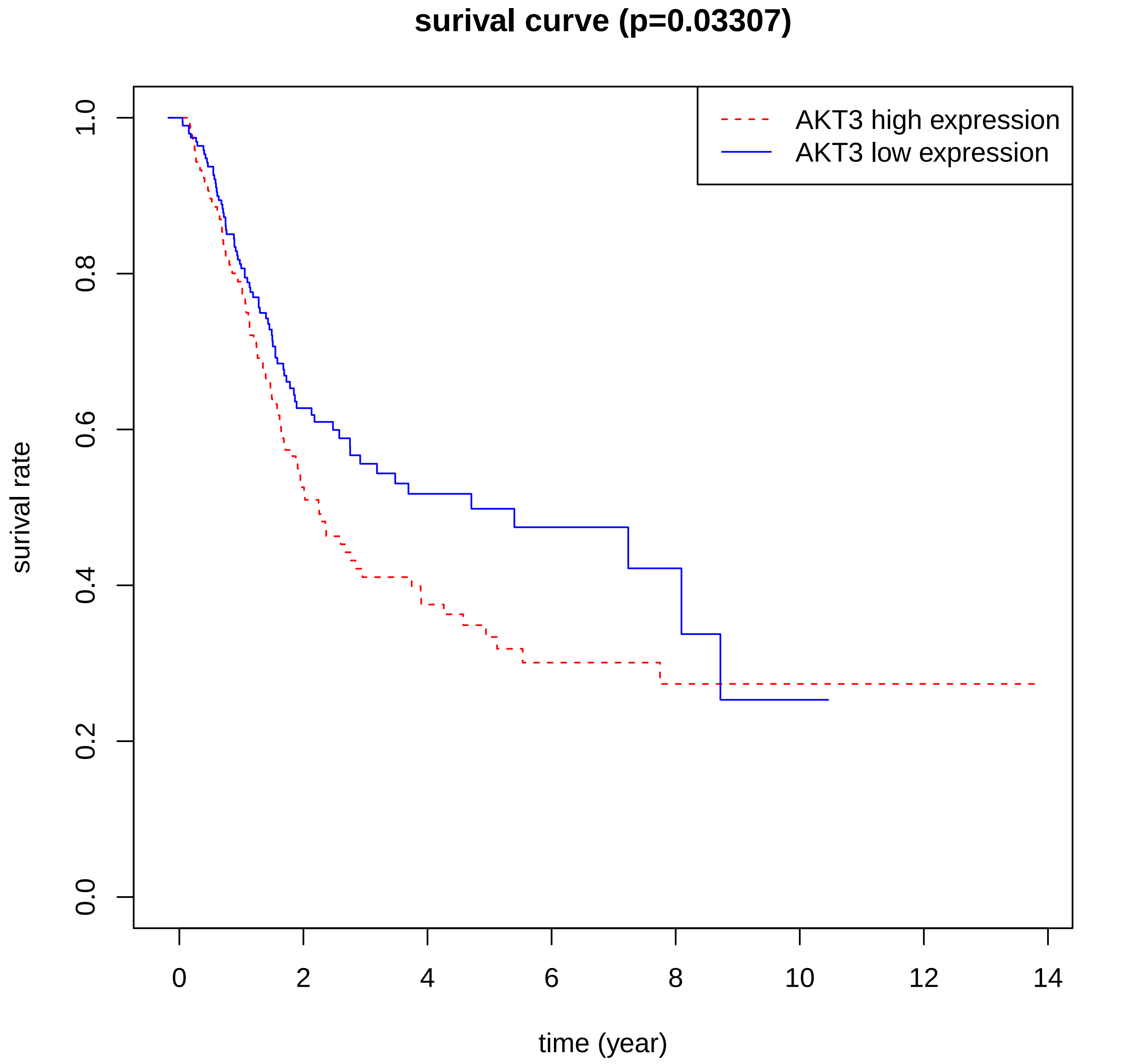
surival curve (p=0.03307)
AKT3 high expression
AKT3 low expression
surival rate
time (year)
0.0
0.2
0.4
0.6
0.8
1.0
0
2
4
6
8
10
12
14

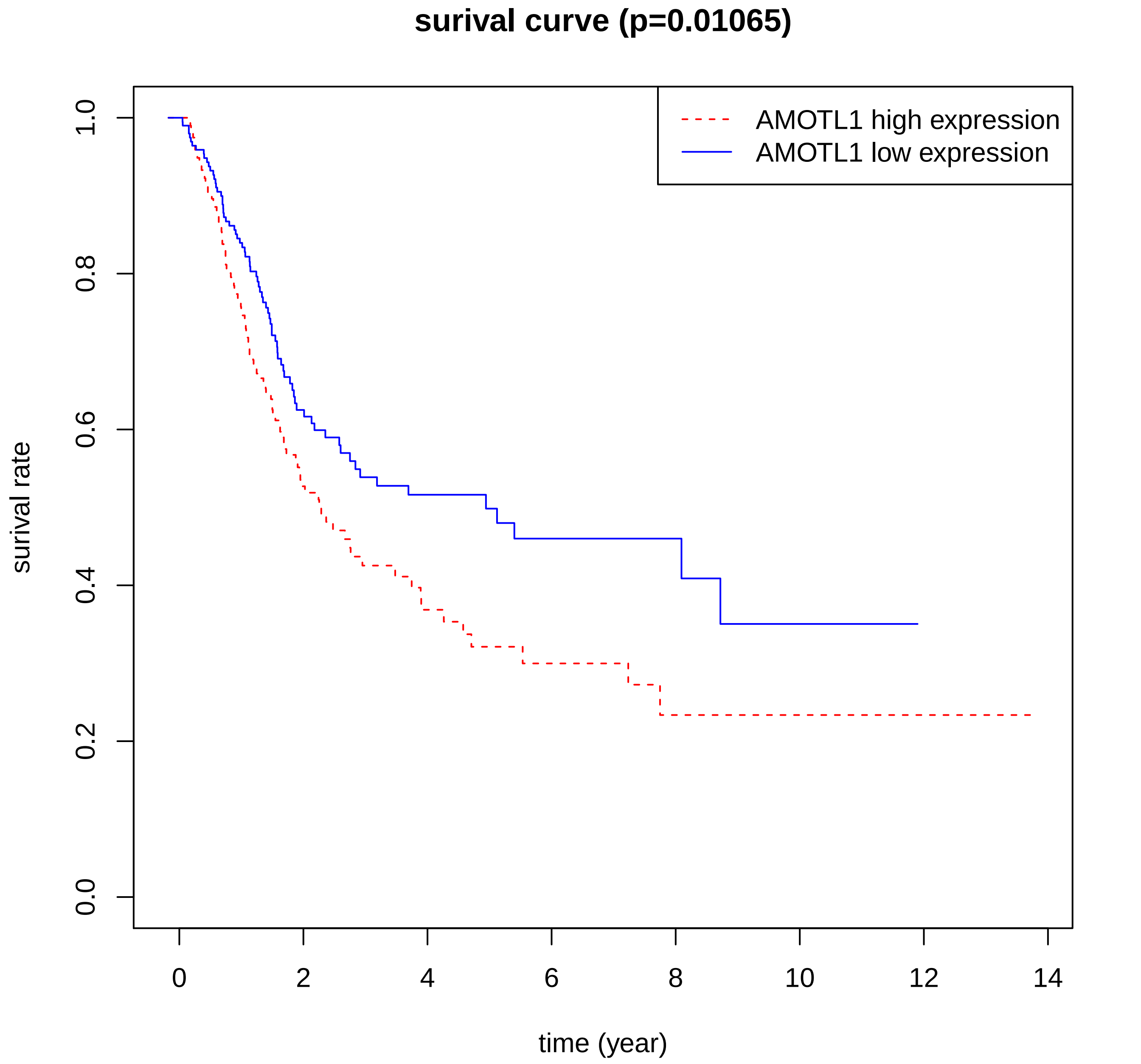

surival curve (p=0.01065)
AMOTL1 high expression
AMOTL1 low expression
surival rate
0.0
0.2
0.4
0.6
0.8
1.0
0
2
4
6
8
10
12
14
time (year)

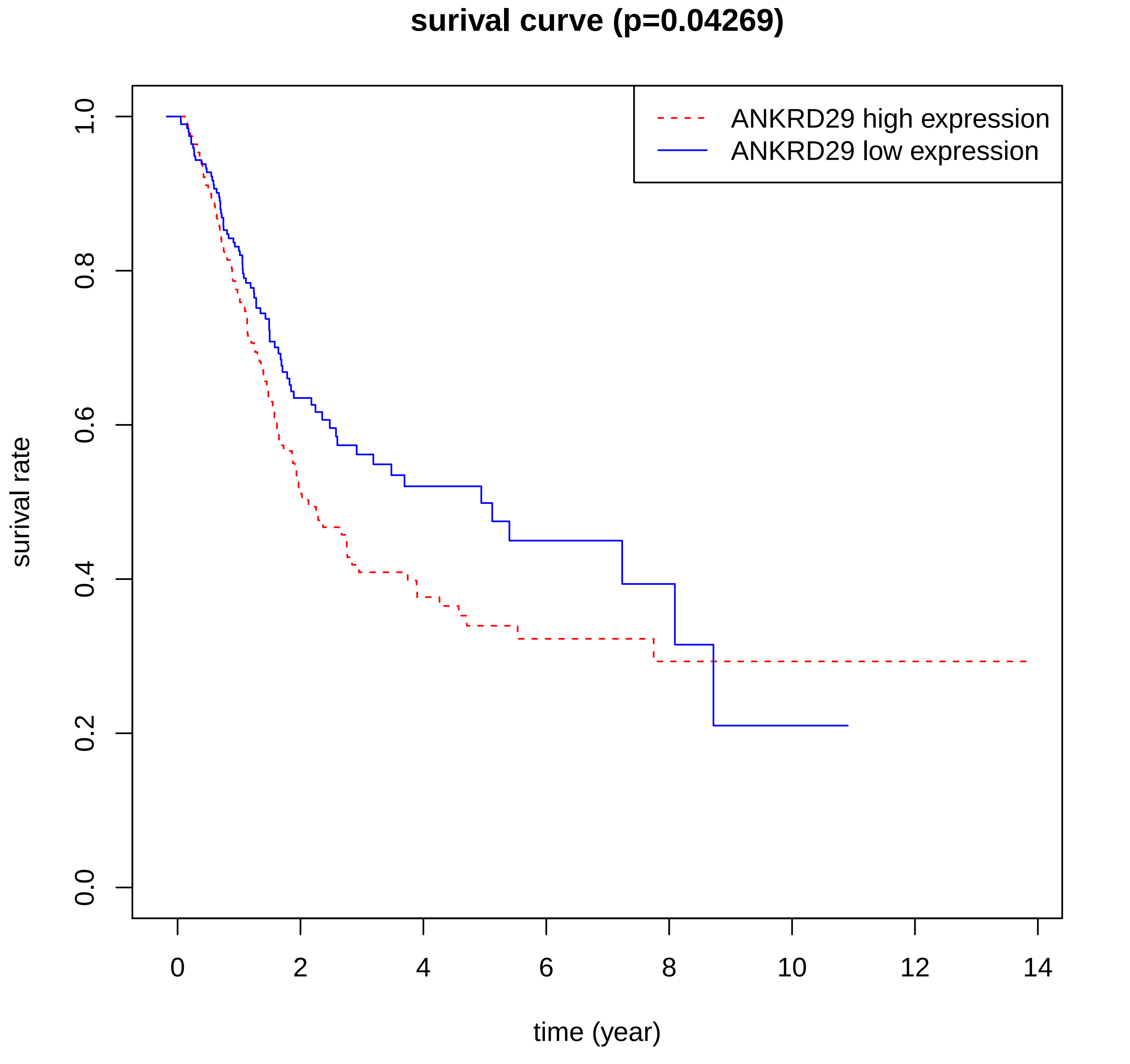
surival curve (p=0.04269)
ANKRD29 high expression
ANKRD29 low expression
surival rate
time (year)
0.0
0.2
0.4
0.6
0.8
1.0
0
2
4
6
8
10
12
14

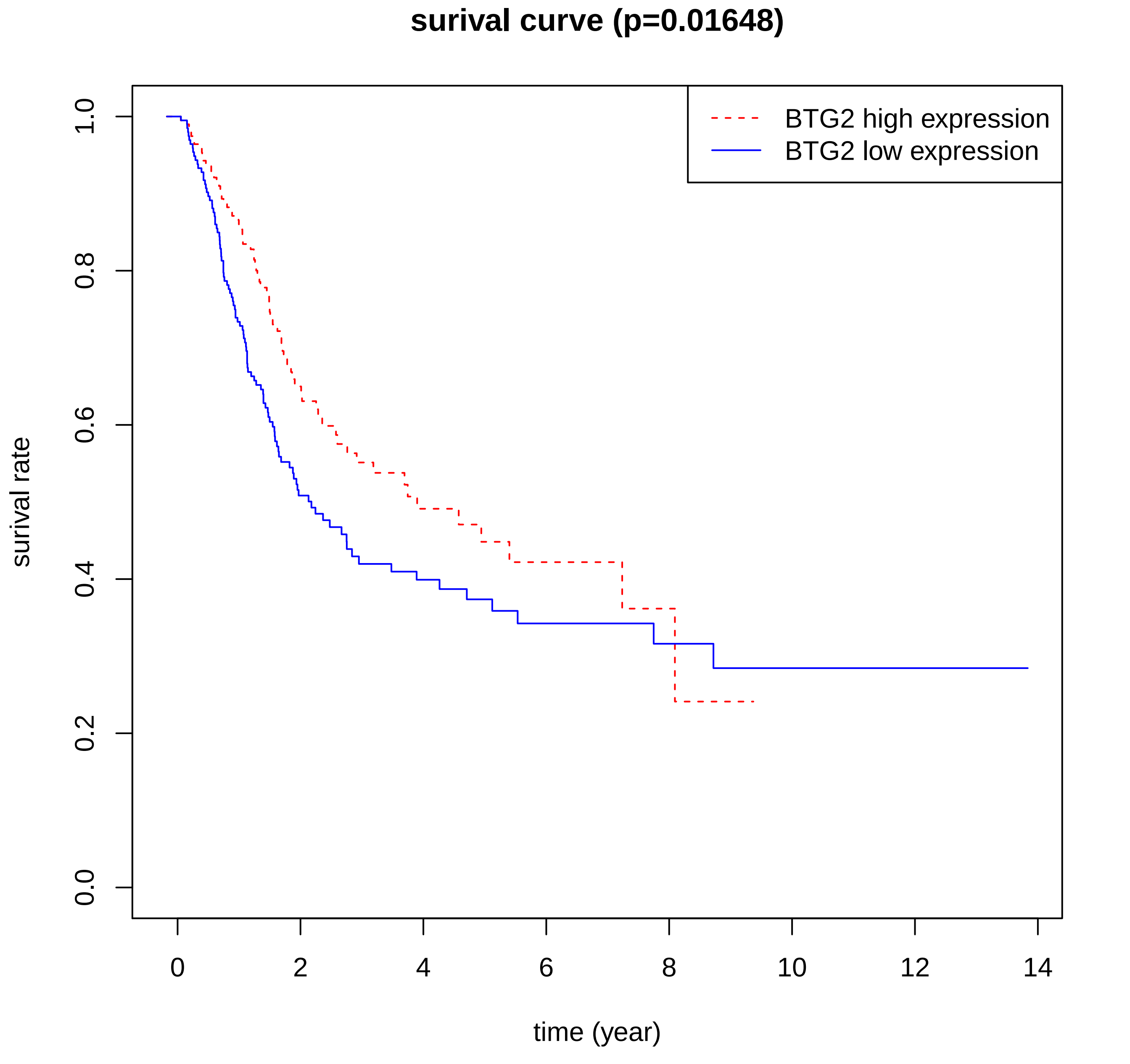

surival curve (p=0.01648)
BTG2 high expression
BTG2 low expression
surival rate
0.0
0.2
0.4
0.6
0.8
1.0
0
2
4
6
8
10
12
14
time (year)

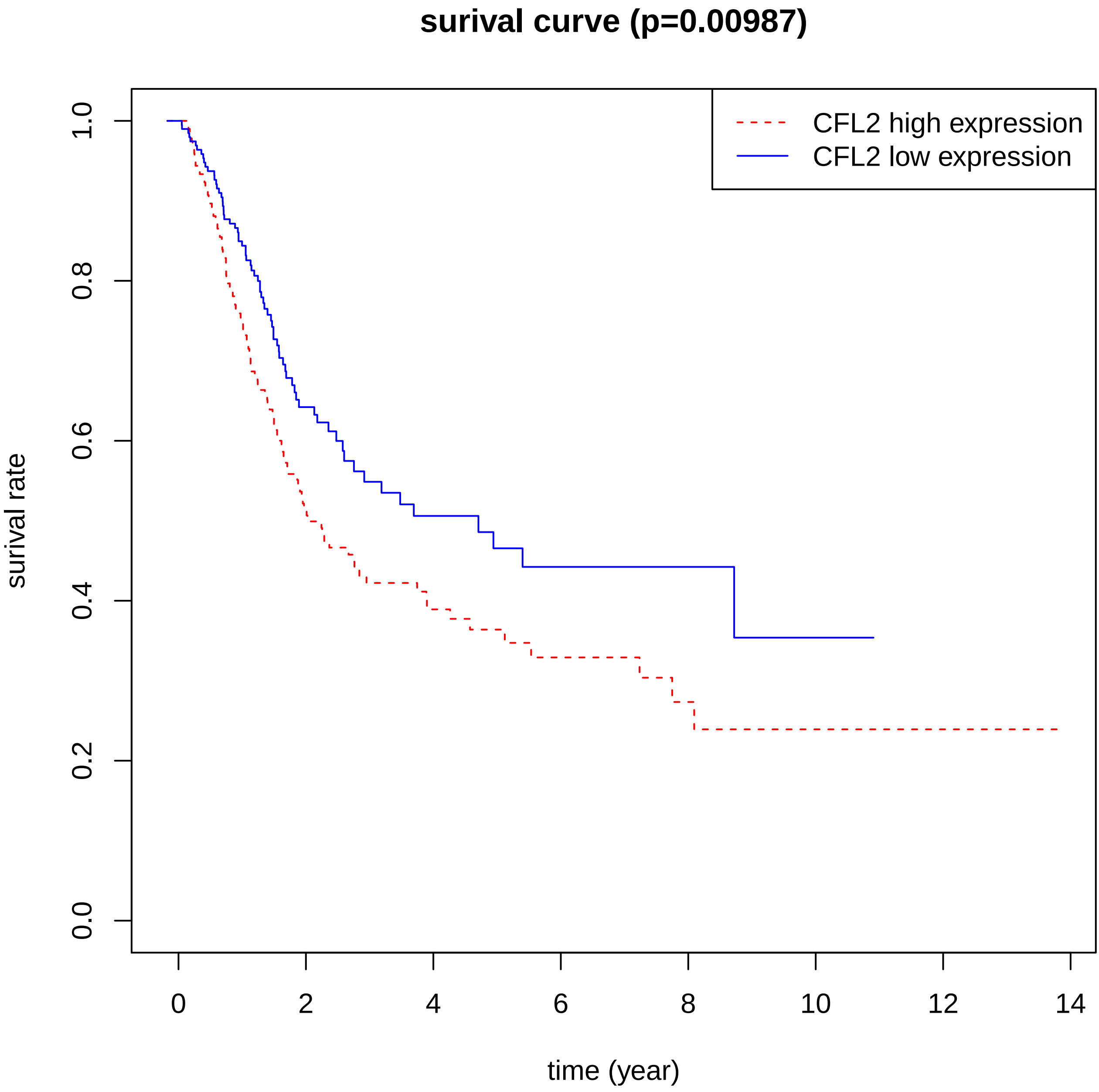

surival curve (p=0.00987)
CFL2 high expression
CFL2 low expression
surival rate
0.0
0.2
0.4
0.6
0.8
1.0
0
2
4
6
8
10
12
14
time (year)

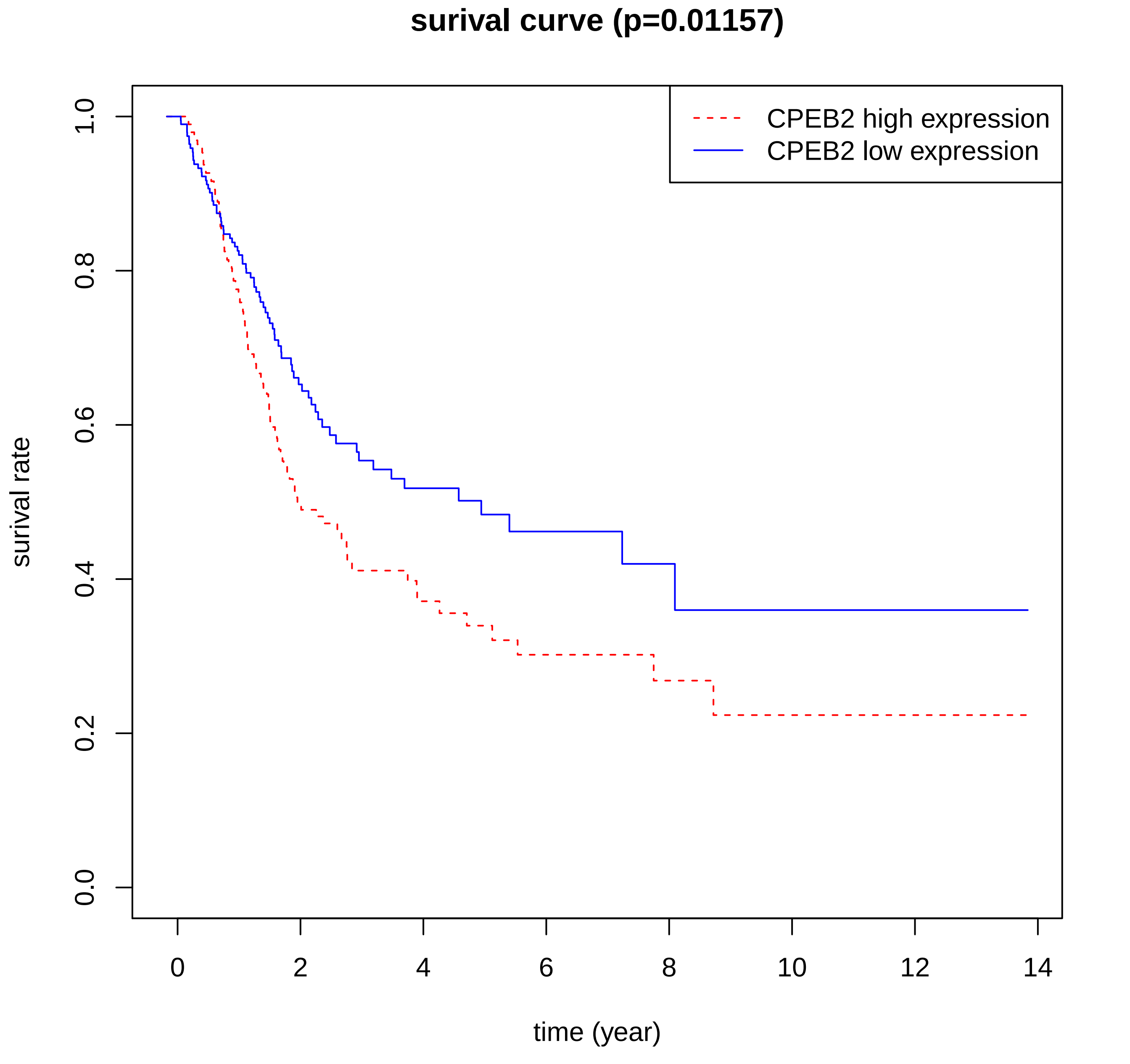
surival curve (p=0.01157)
CPEB2 high expression
CPEB2 low expression
surival rate
1.0
0.8
0.6
0.4
0.2
0.0
0
2
4
6
8
10
12
14
time (year)

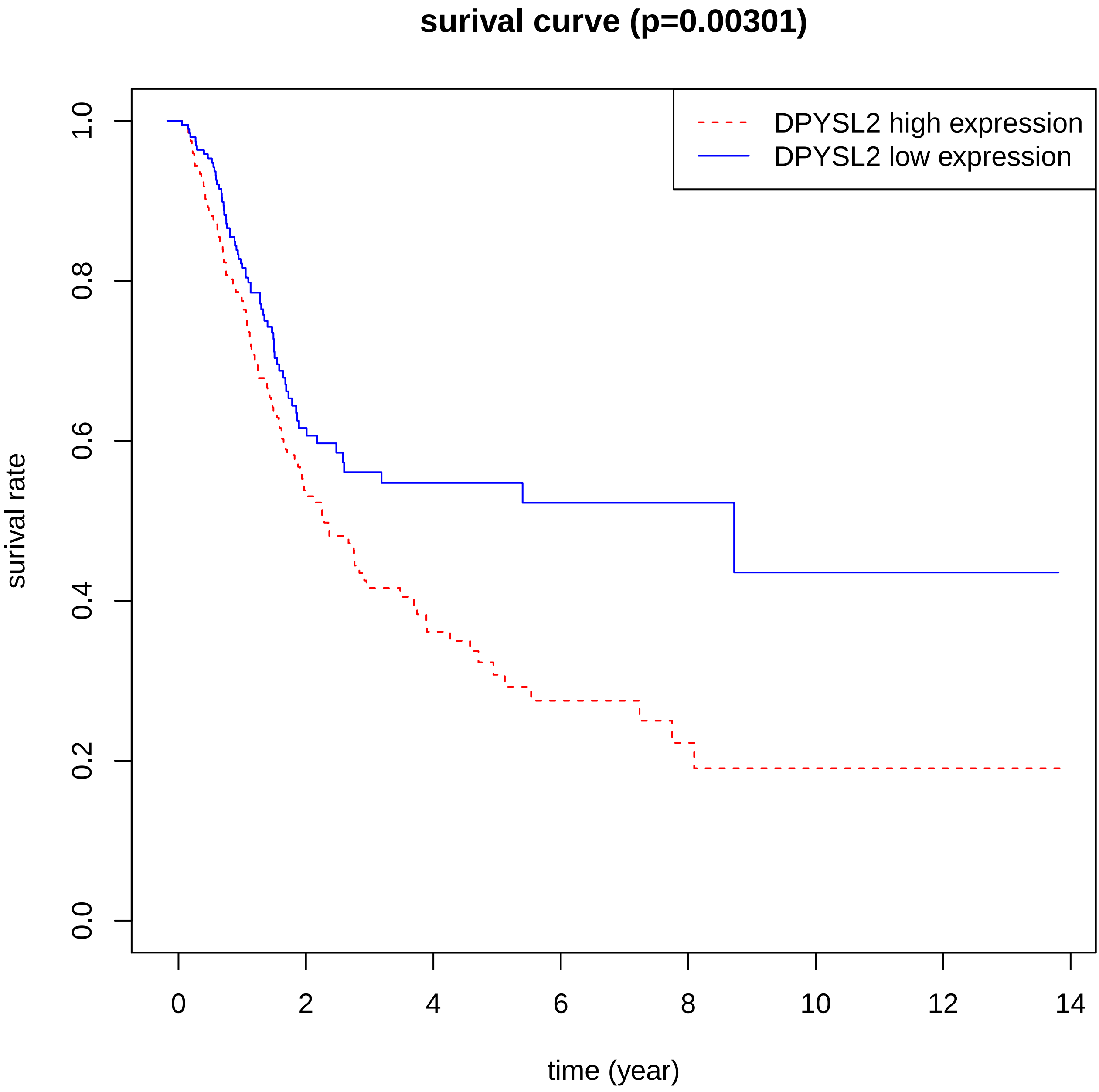
surival curve (p=0.00301)
DPYSL2 high expression
DPYSL2 low expression
surival rate
1.0
0.8
0.6
0.4
0.2
0.0
0
2
4
6
8
10
12
14
time (year)

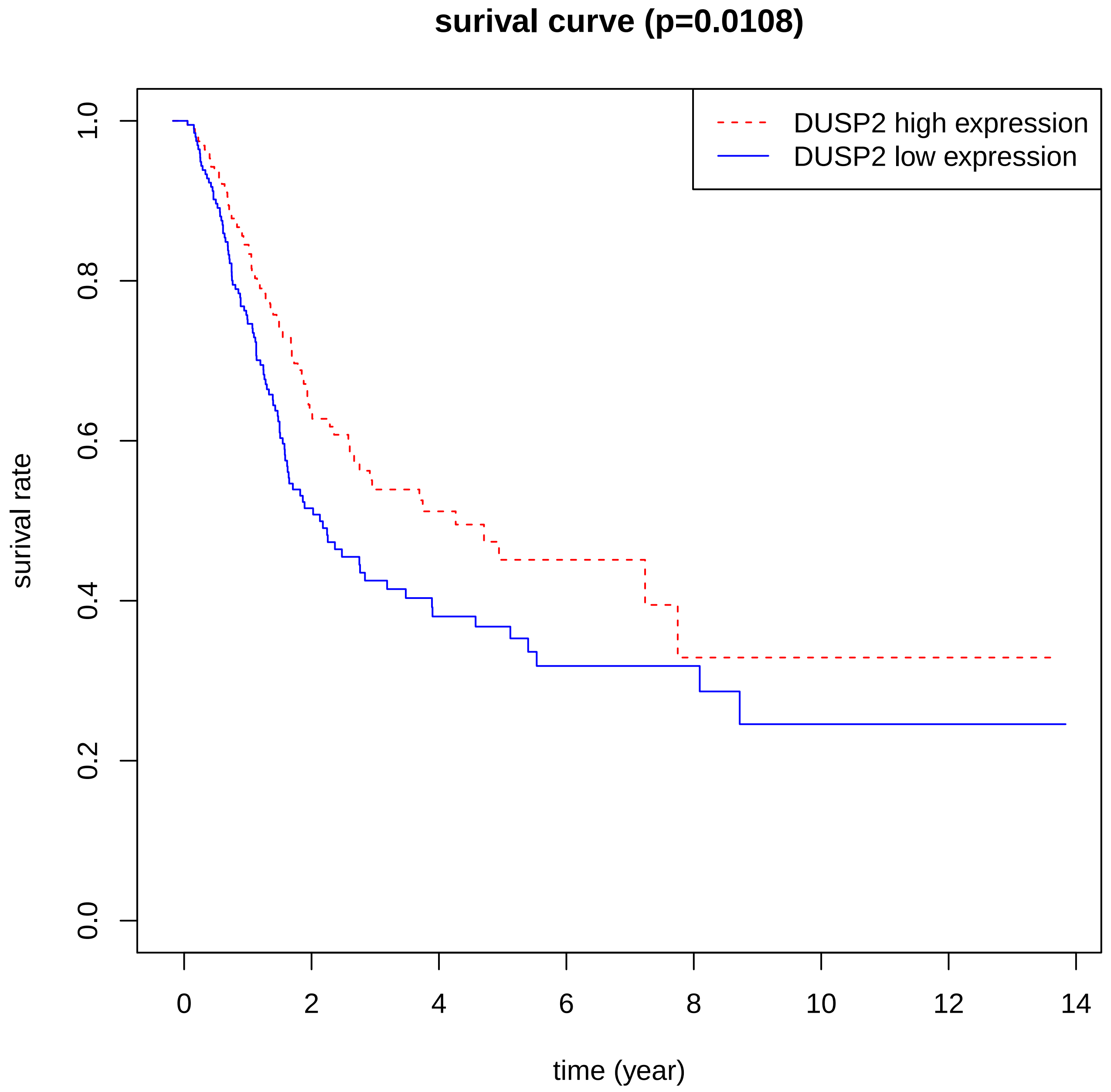

surival curve (p=0.0108)
DUSP2 high expression
DUSP2 low expression
surival rate
0.0
0.2
0.4
0.6
0.8
1.0
0
2
4
6
8
10
12
14
time (year)

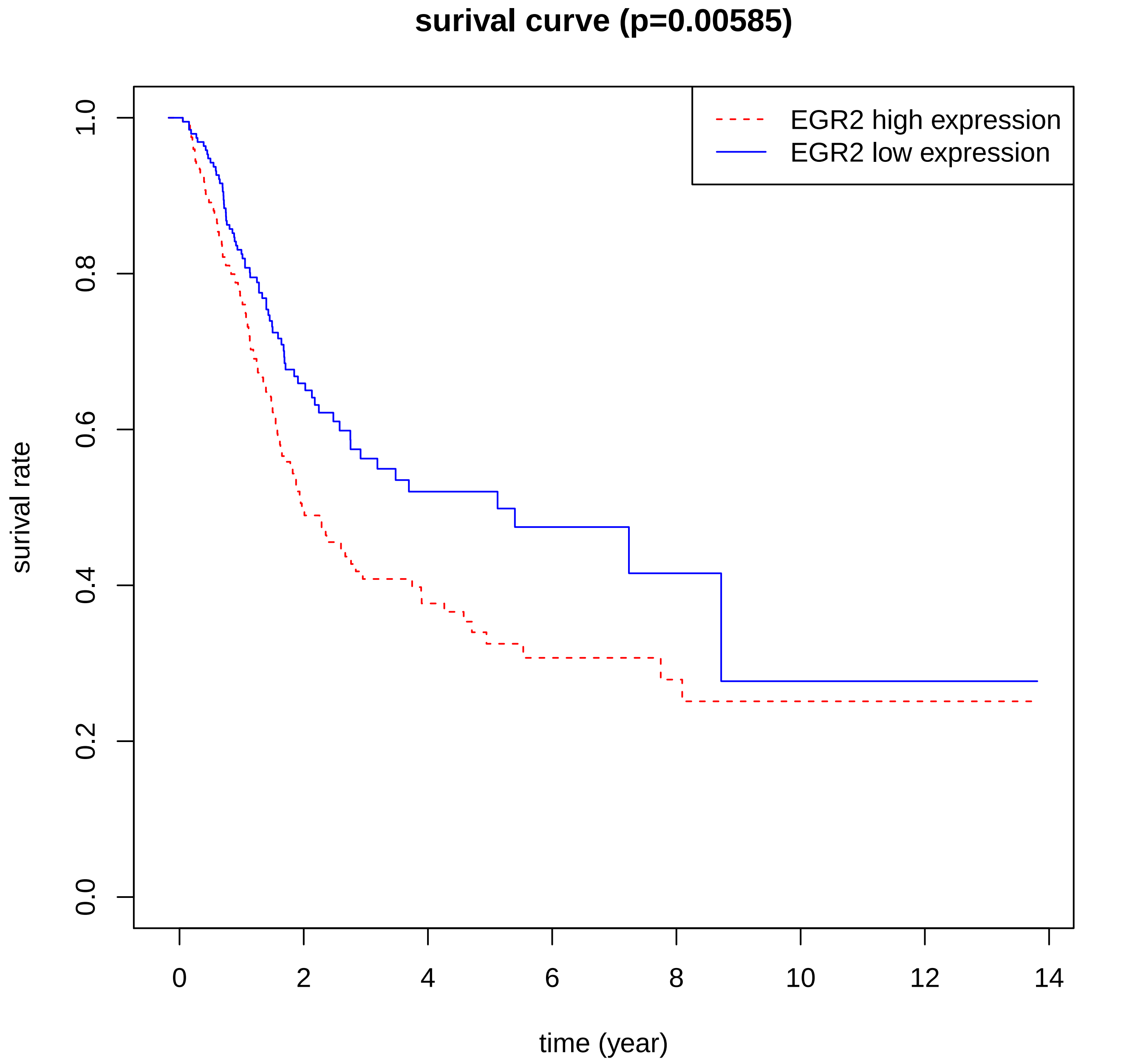

surival curve (p=0.00585)
EGR2 high expression
EGR2 low expression
surival rate
1.0
0.8
0.6
0.4
0.2
0.0
0
2
4
6
8
10
12
14
time (year)

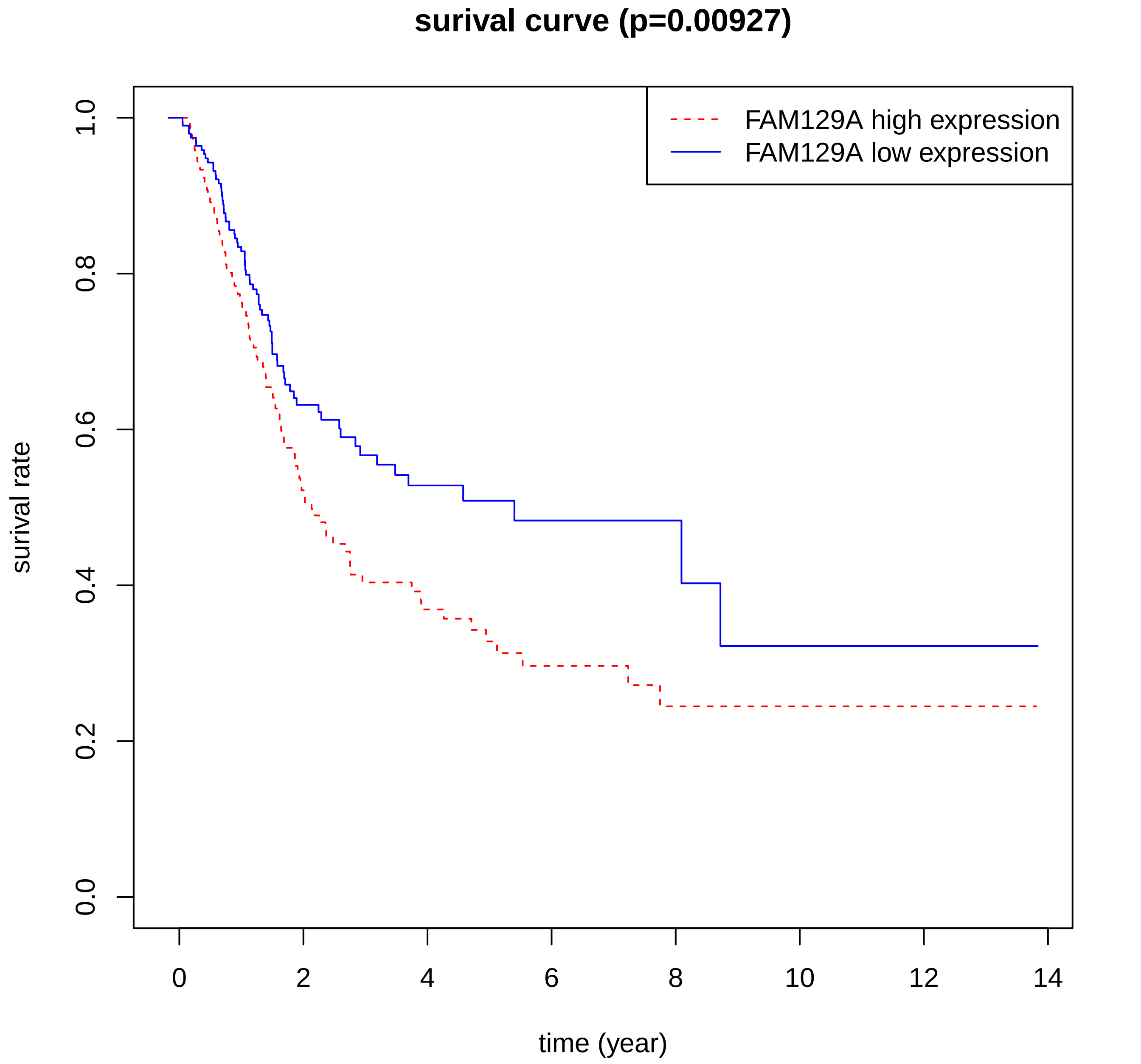
surival curve (p=0.00927)
FAM129A high expression
FAM129A low expression
surival rate
0.0
0.2
0.4
0.6
0.8
1.0
0
2
4
6
8
10
12
14
time (year)

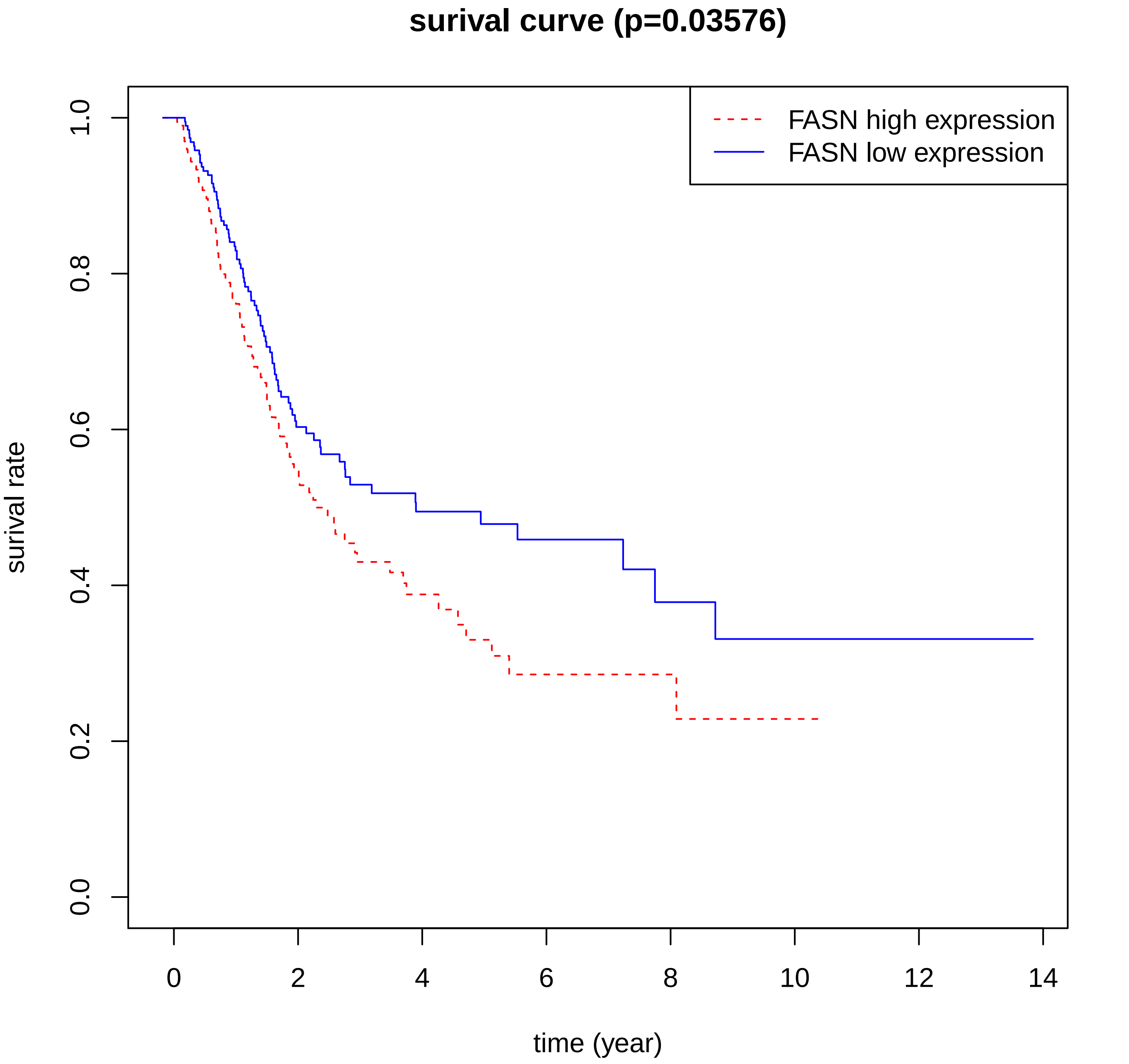
surival curve (p=0.03576)
FASN high expression
FASN low expression
surival rate
0.0
0.2
0.4
0.6
0.8
1.0
time (year)
0
2
4
6
8
10
12
14

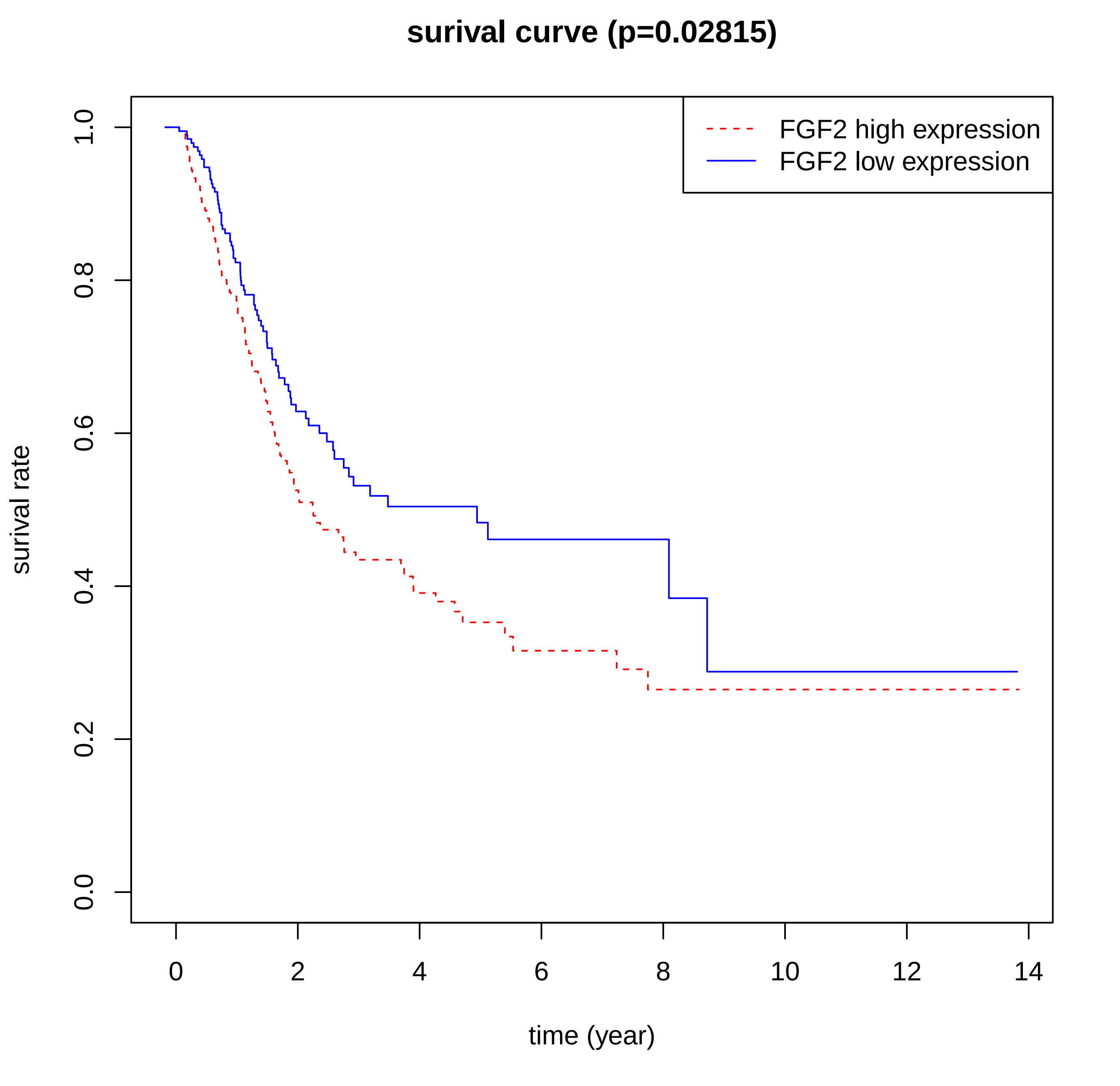

surival curve (p=0.02815)
FGF2 high expression
FGF2 low expression
surival rate
time (year)
0.0
0.2
0.4
0.6
0.8
1.0
0
2
4
6
8
10
12
14

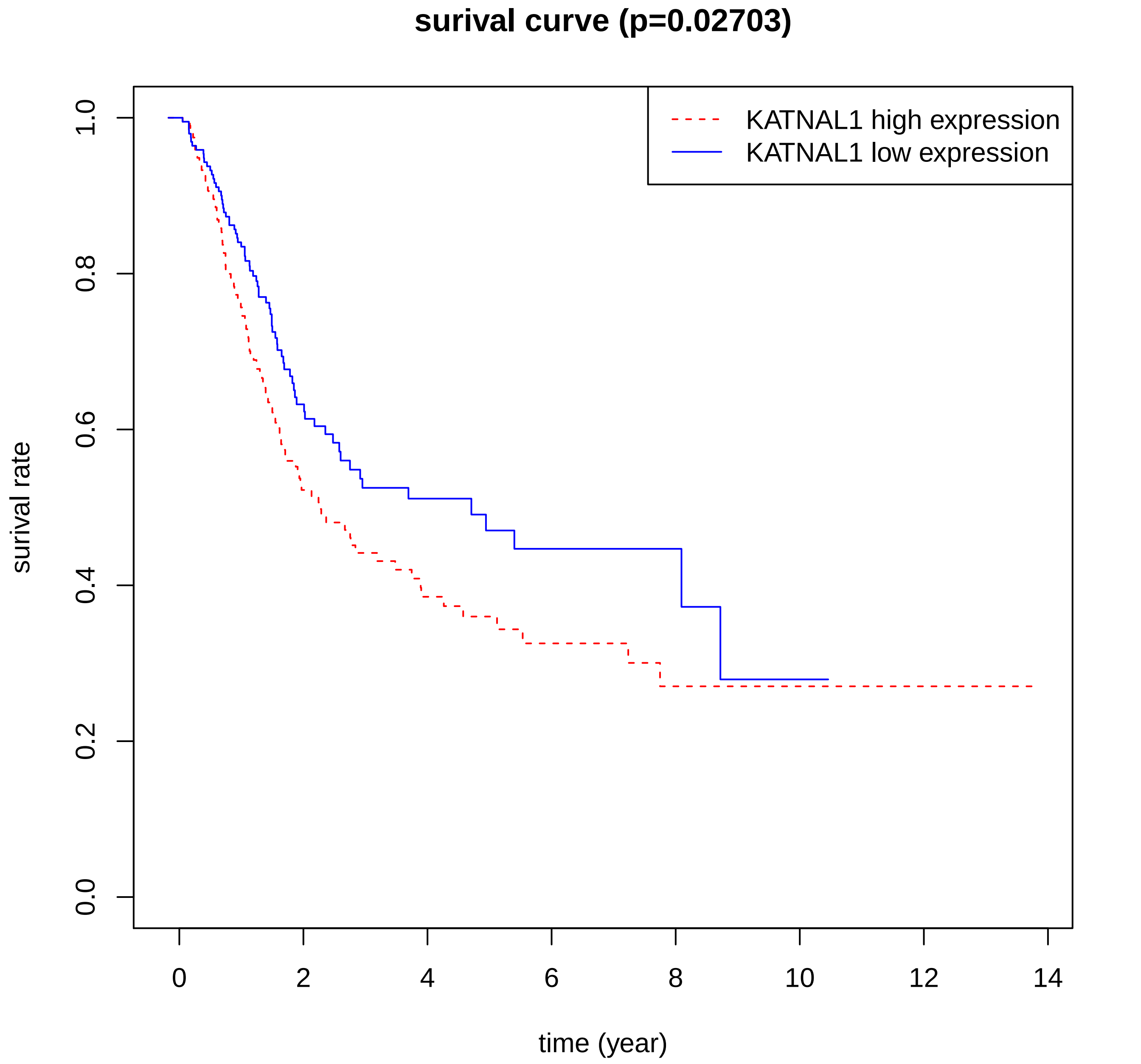

surival curve (p=0.02703)
KATNAL1 high expression
KATNAL1 low expression
surival rate
time (year)
0.0
0.2
0.4
0.6
0.8
1.0
0
2
4
6
8
10
12
14

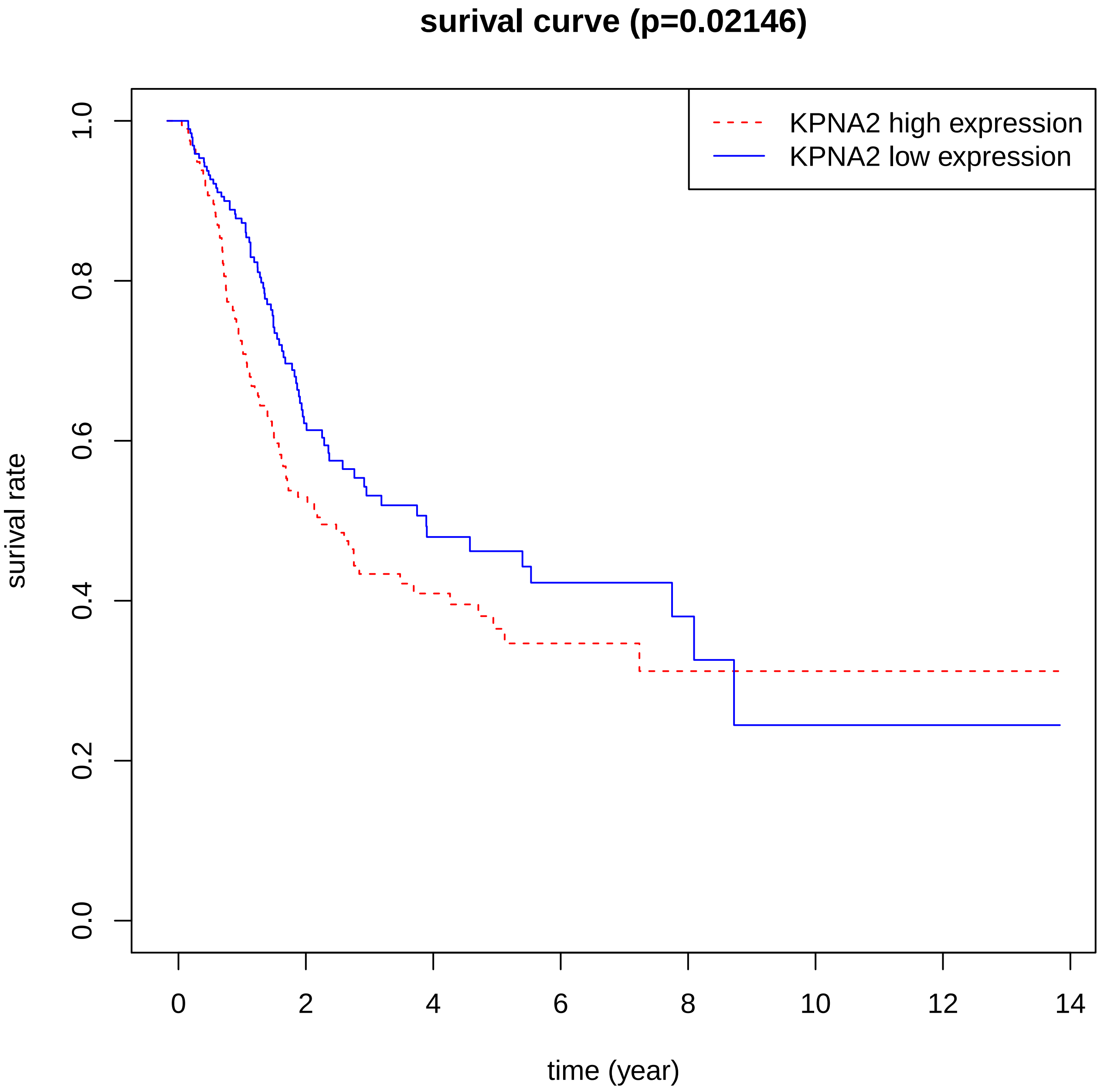

surival curve (p=0.02146)
KPNA2 high expression
KPNA2 low expression
1.0
0.8
0.6
0.4
0.2
0.0
surival rate
0
2
4
6
8
10
12
14
time (year)

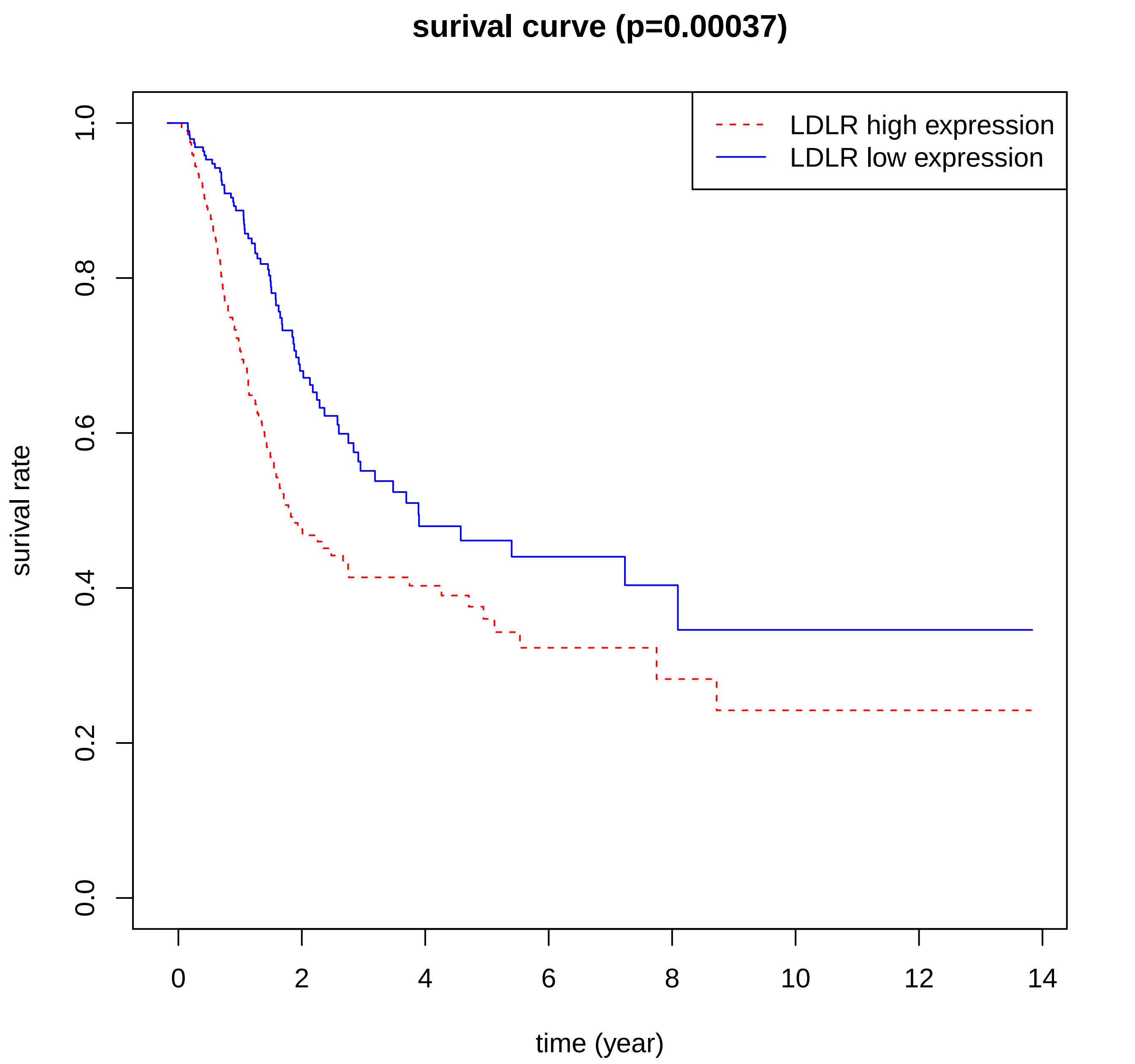
surival curve (p=0.00037)
LDLR high expression
LDLR low expression
surival rate
time (year)
0.0
0.2
0.4
0.6
0.8
1.0
0
2
4
6
8
10
12
14

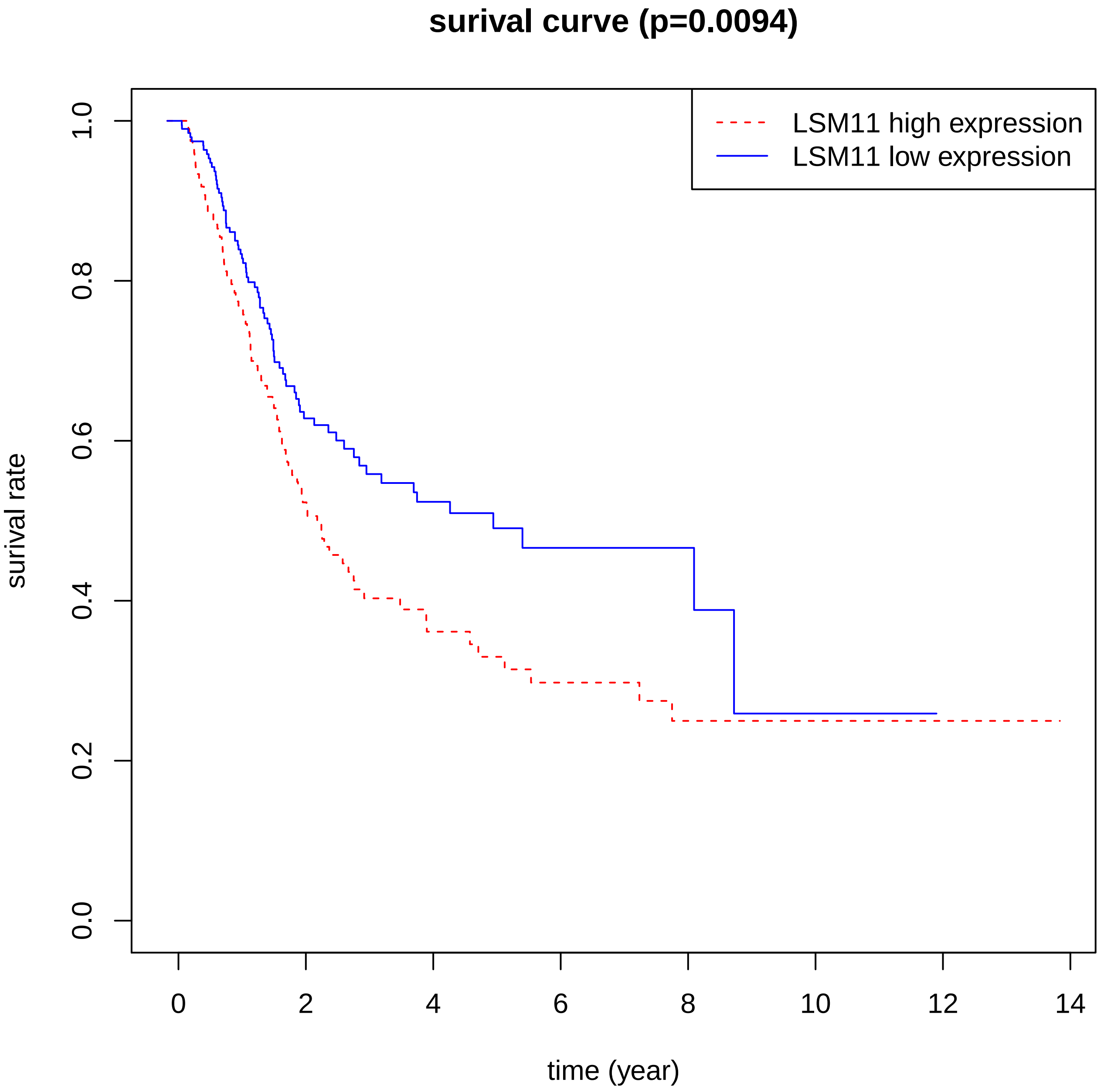

surival curve (p=0.0094)
LSM11 high expression
LSM11 low expression
surival rate
time (year)
0.0
0.2
0.4
0.6
0.8
1.0
0
2
4
6
8
10
12
14

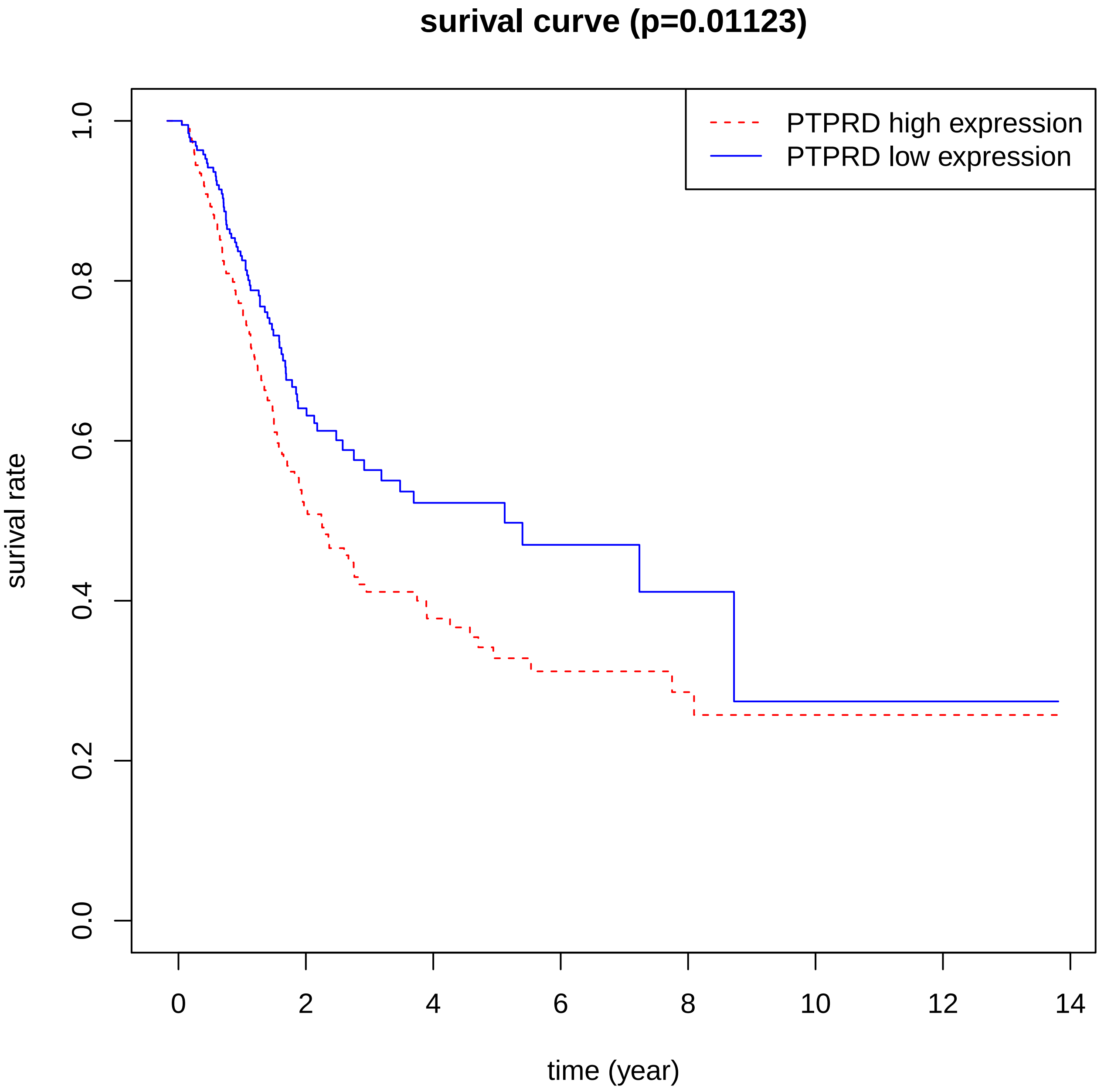
surival curve (p=0.01123)
PTPRD high expression
PTPRD low expression
surival rate
time (year)
0.0
0.2
0.4
0.6
0.8
1.0
0
2
4
6
8
10
12
14

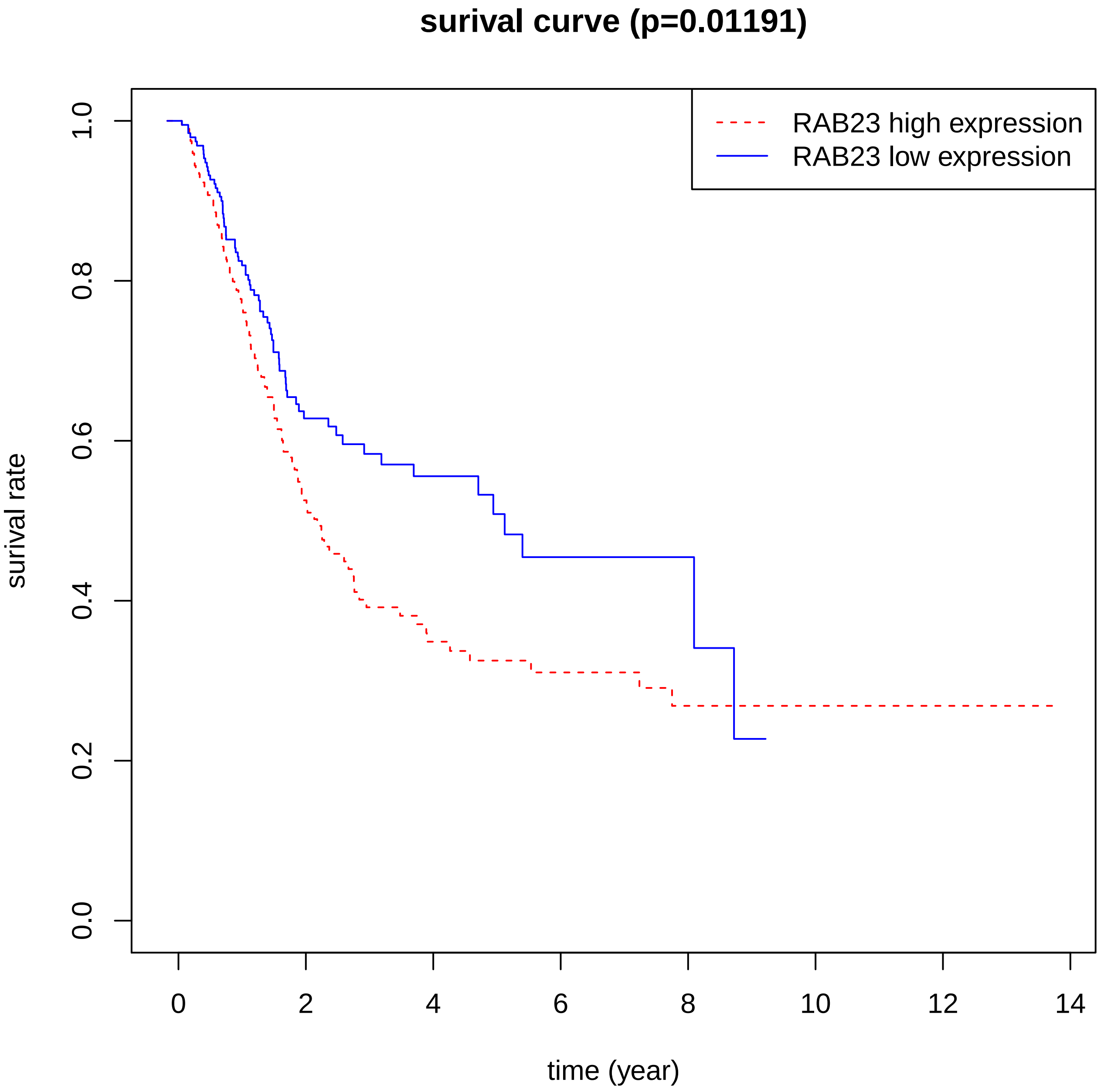
surival curve (p=0.01191)
RAB23 high expression
RAB23 low expression
surival rate
time (year)
0.0
0.2
0.4
0.6
0.8
1.0
0
2
4
6
8
10
12
14

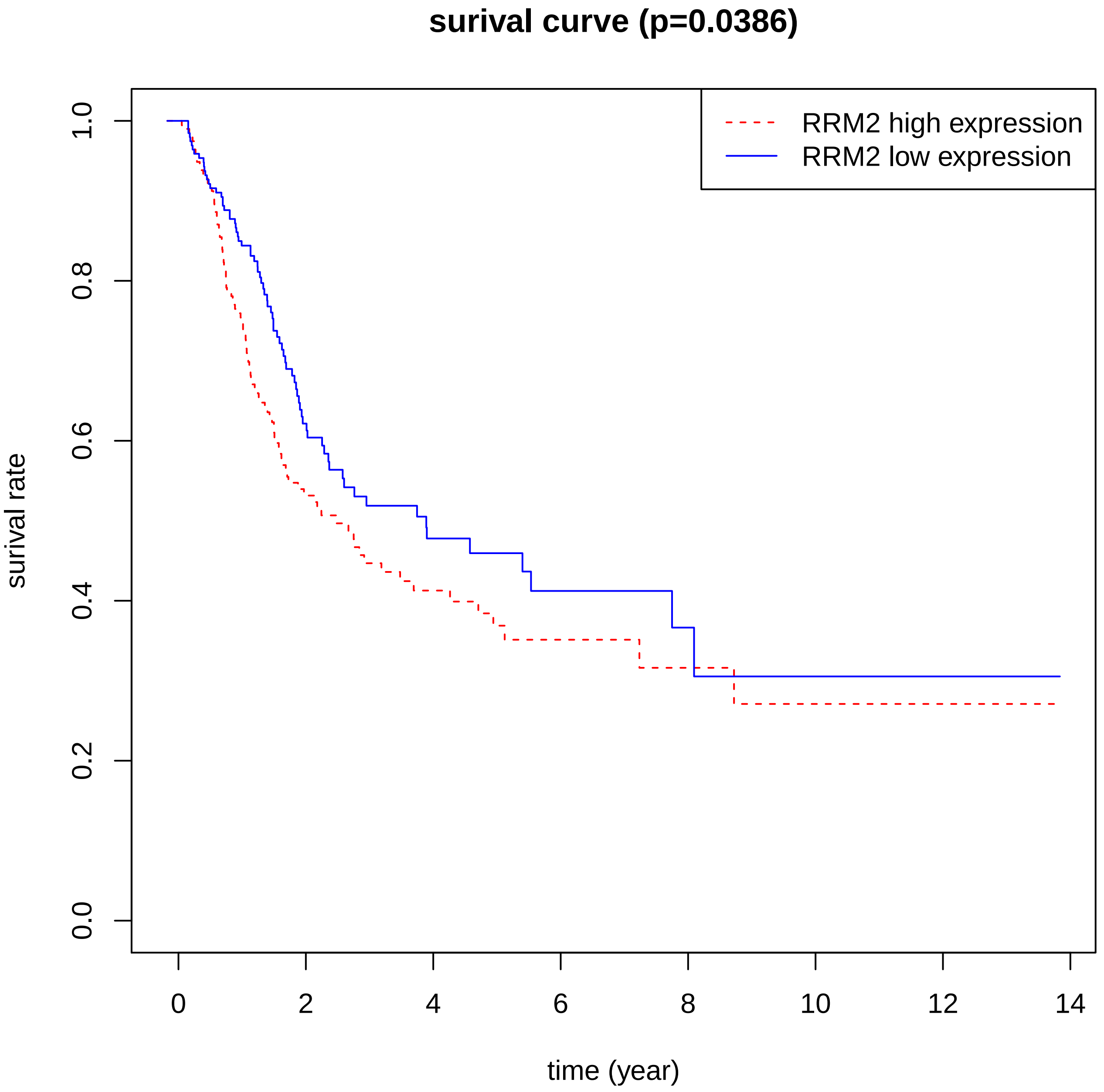

surival curve (p=0.0386)
RRM2 high expression
RRM2 low expression
surival rate
0.0
0.2
0.4
0.6
0.8
1.0
0
2
4
6
8
10
12
14
time (year)

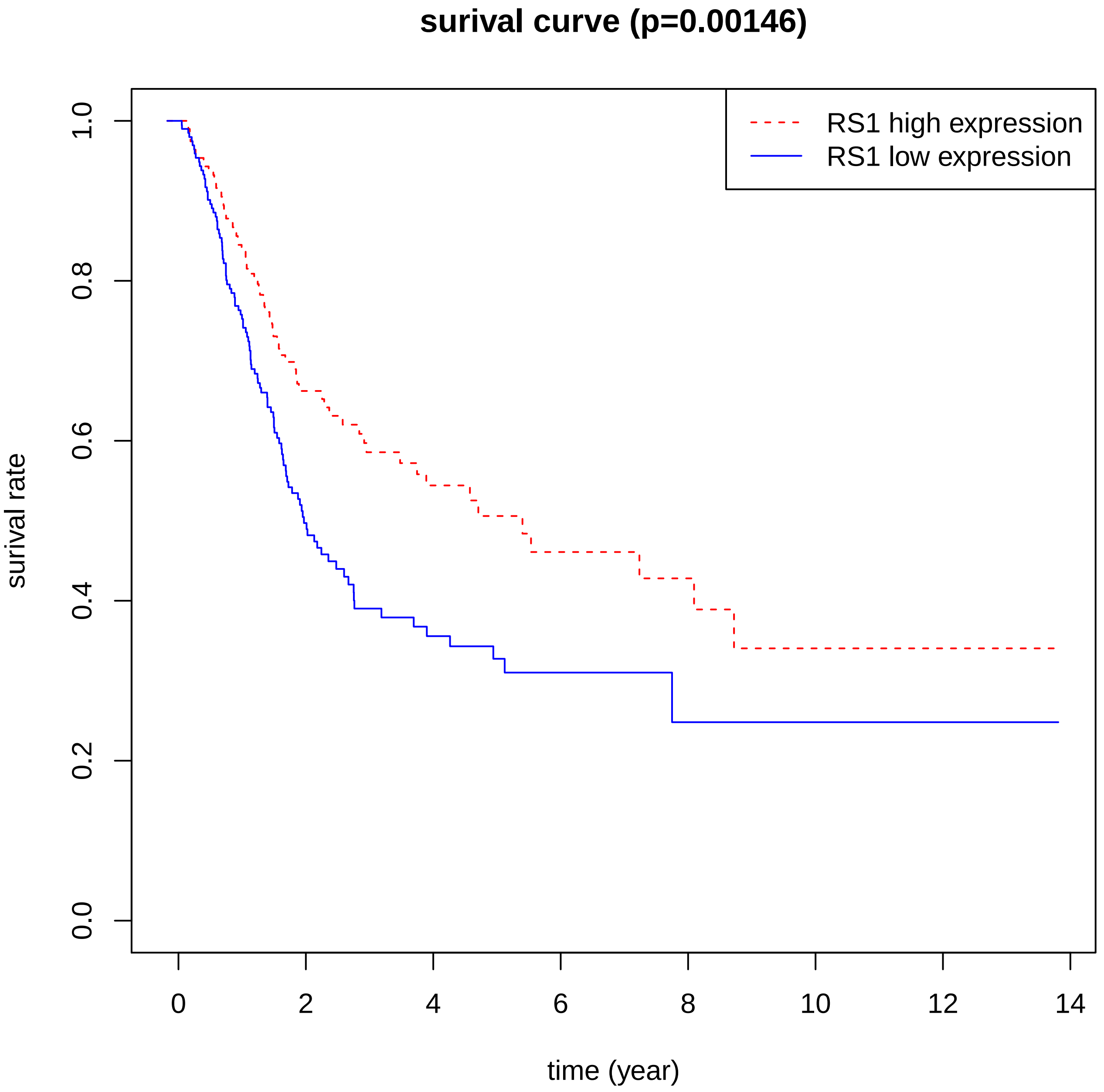

surival curve (p=0.00146)
RS1 high expression
RS1 low expression
surival rate
time (year)
0.0
0.2
0.4
0.6
0.8
1.0
0
2
4
6
8
10
12
14

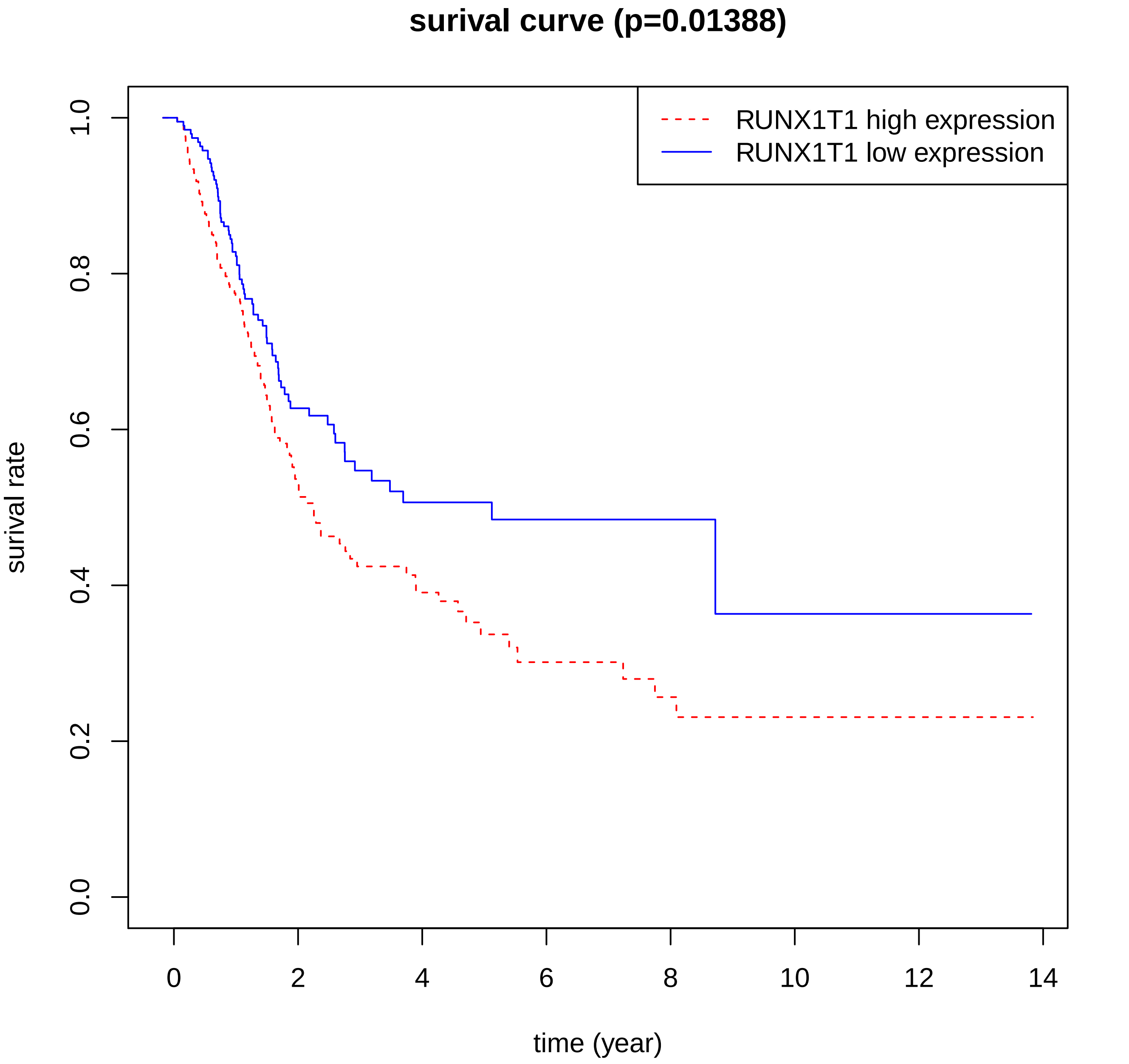

surival curve (p=0.01388)
RUNX1T1 high expression
RUNX1T1 low expression
surival rate
0.0
0.2
0.4
0.6
0.8
1.0
0
2
4
6
8
10
12
14
time (year)

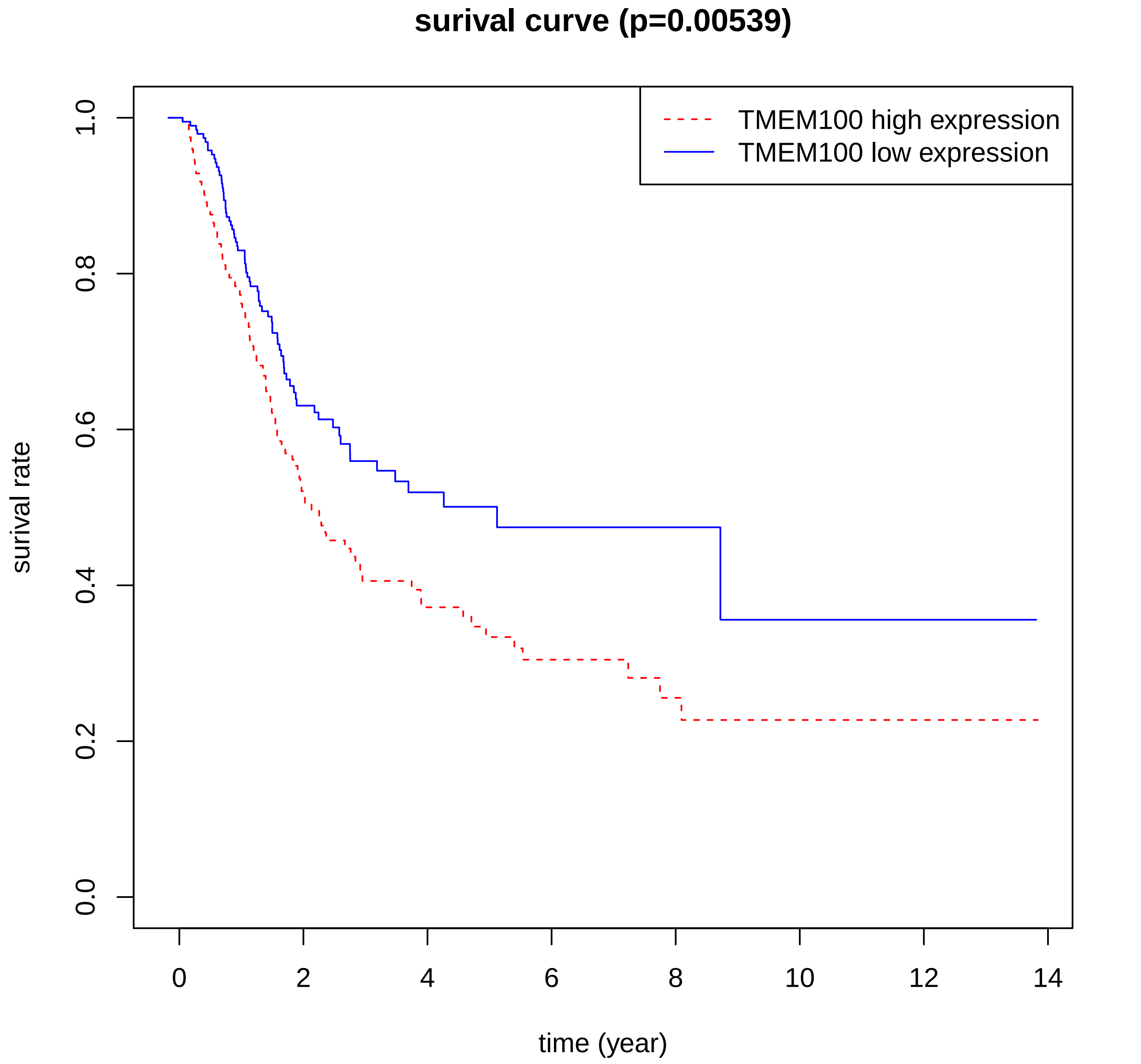

surival curve (p=0.00539)
TMEM100 high expression
TMEM100 low expression
surival rate
0.0
0.2
0.4
0.6
0.8
1.0
time (year)
0
2
4
6
8
10
12
14

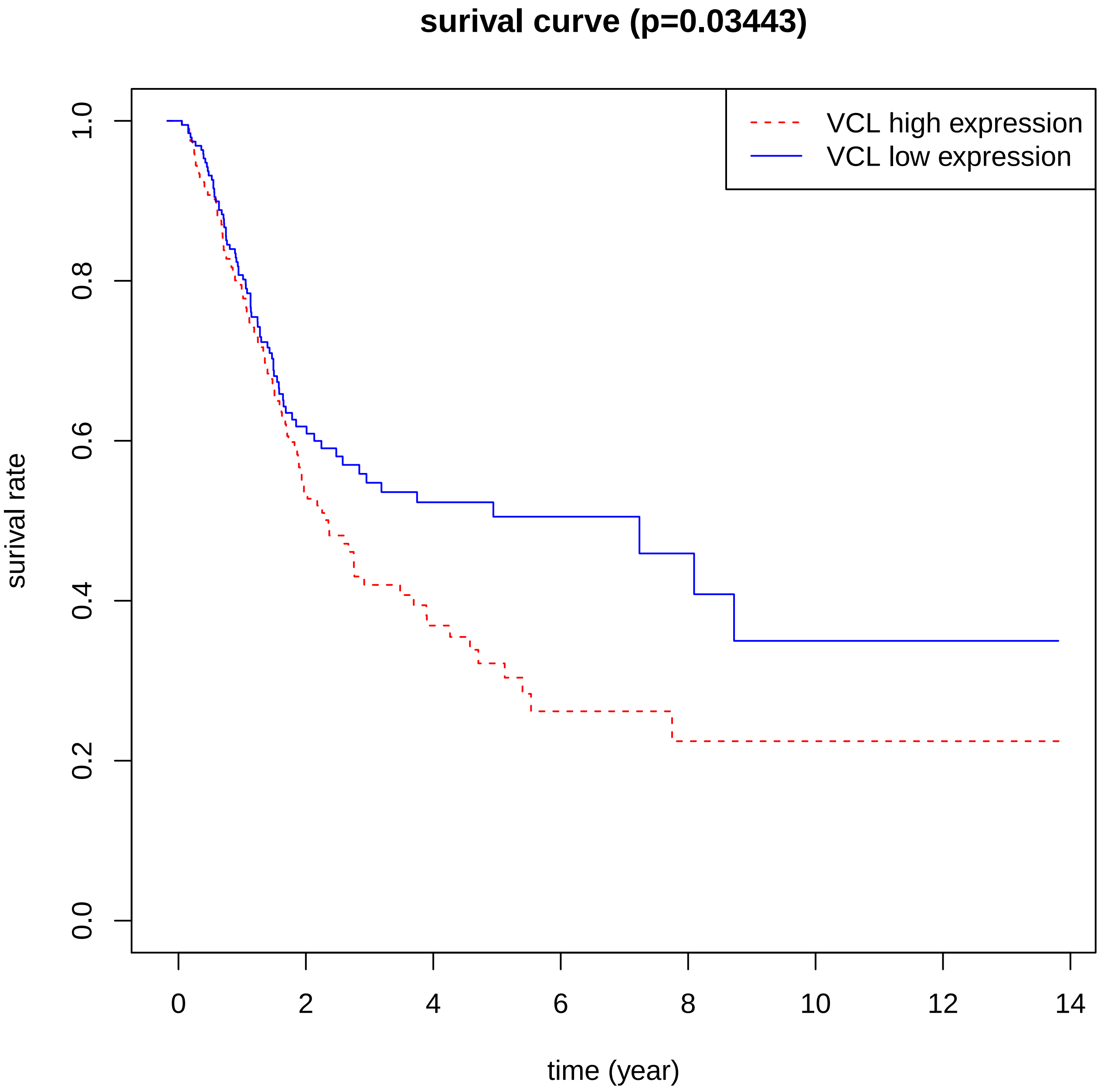

surival curve (p=0.03443)
VCL high expression
VCL low expression
surival rate
1.0
0.8
0.6
0.4
0.2
0.0
0
2
4
6
8
10
12
14
time (year)

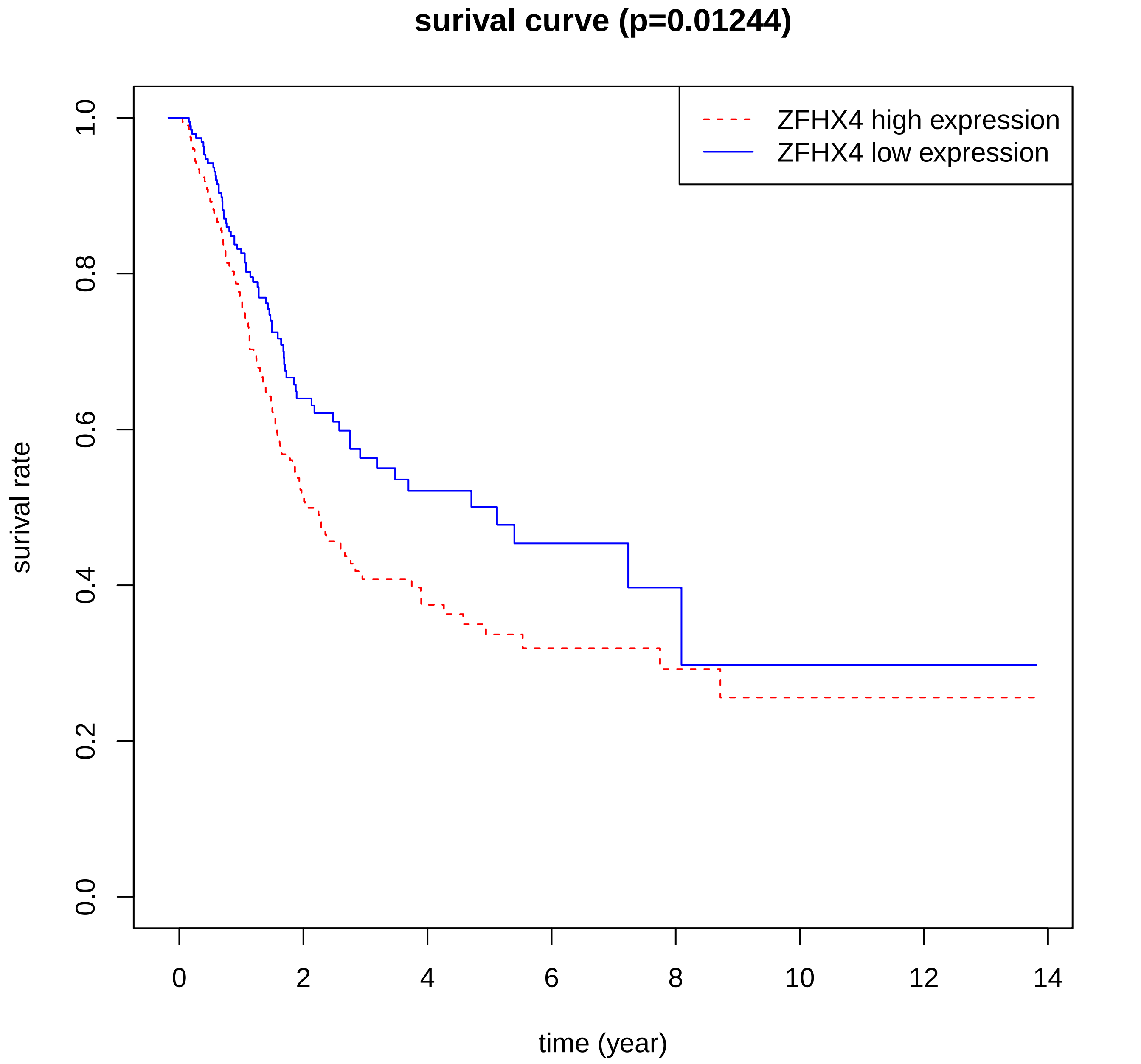
surival curve (p=0.01244)
ZFHX4 high expression
ZFHX4 low expression
surival rate
0.0
0.2
0.4
0.6
0.8
1.0
0
2
4
6
8
10
12
14
time (year)
